# Therapies and delivery systems for diabetic wound care: current insights and future directions

**DOI:** 10.3389/fphar.2025.1628252

**Published:** 2025-07-22

**Authors:** Preeti Singh Yadav, Mahendra Singh, Ramachandran Vinayagam, Prashant Shukla

**Affiliations:** ^1^ Department of Pharmaceutics, Hygia Institute of Pharmacy, Lucknow, Uttar Pradesh, India; ^2^ Department of Biotechnology, College of Life and Applied Sciences, Yeungnam University, Gyeongsan, Gyeongsangbuk-do, Republic of Korea; ^3^ Department of Pharmaceutical Sciences, School of Health Sciences and Technology UPES, Dehradun, Uttarakhand, India

**Keywords:** antioxidants, hyperglycemia, phytopharmaceuticals, nanotechnology, topical therapy

## Abstract

The global rise in diabetes mellitus has been paralleled by an increase in associated complications, notably impaired Wound Healing. Non-healing diabetic wounds are driven by multifactorial pathogenesis involving hyperglycemia, immune dysfunction, impaired angiogenesis, bacterial infections, and increased oxidative stress. Traditionally, a variety of plant-derived extracts and phytochemicals such as quercetin, curcumin, and paeoniflorin have been employed in the treatment of diabetic wounds worldwide. These agents exert their therapeutic effects primarily through antioxidant, antibacterial, anti-inflammatory, and pro-angiogenic mechanisms and properties, typically with minimal side effects. Recent advancements have highlighted the potential of integrating phytoconstituents with metal nanoparticles to enhance Wound Healing efficacy. Nanoformulations improve targeted phytochemical delivery and offer synergistic benefits due to intrinsic antimicrobial and antioxidant properties, enhanced antioxidant activity, and high biocompatibility. Similarly, polymeric nanocarrier-based delivery systems have emerged as a promising strategy to address the limitations of conventional wound treatments, promoting faster and more efficient healing in diabetic patients. This review comprehensively discusses the pathophysiology and clinical challenges associated with diabetic Wound Healing, explores the therapeutic potential of key phytochemicals, and presents the current progress in nanoparticle-based delivery systems (metallic and polymeric) for diabetic wound management. Additionally, it provides an update on recent patents and clinical trials involving phytoconstituents and their formulations for the treatment of diabetic wounds.

## 1 Introduction

Diabetes is a chronic disease that occurs from either insufficient insulin production by the pancreas or ineffective insulin action by the body (Sultana et al., 2025). Uncontrolled diabetes leads to hyperglycemia, which over time damages the kidneys, arteries, nerves, and causes cancer-related issues (McDermott et al., 2023). Hyperglycemia is caused by the malfunctioning or destruction of the pancreas and insulin-producing β-cells, leading to inadequate insulin secretion.

Compared to high-income countries, diabetes is rising more quickly in low- and middle-income countries. Recently, it was projected by the International Diabetes Federation that 10% of the global population suffered from diabetes in 2021; by 2030 and 2045, that number is expected to rise to 643 million and 783 million, respectively (Monteiro-Soares and Santos, 2023; Khattak et al., 2025). In 2021, kidney disease due to diabetes caused over 2 million deaths (WHO, 2024). Additionally, it was anticipated to a rise in diabetes cases globally due to modern lifestyles, obesity, physical inactivity, and population growth. This can lead to several serious long-term complications, for instance, neuropathy, nephropathy, retinopathy, cardiovascular diseases, and skin ulcers (Singh et al., 2025). Delayed or nonhealing wounds are among the major consequences associated with hyperglycemia. Many factors can delay healing mechanisms, for example, age-related changes in normal physiological function and unfavorable environmental conditions (Patel et al., 2019). Patients with diabetes may sustain relatively modest wounds, but these wounds might develop into chronic, nonhealing ulcers that cause further infection, gangrene, and occasionally amputation. The highest amputation rate has been reported in diabetic patients (Greenhalgh, 2003). Also, long-term elevated blood glucose levels are common among diabetics, and this can seriously harm the brain, blood vessels, and immune system (Li et al., 2022a; Yuan et al., 2022). Furthermore, chronic inflammation is more likely to develop in diabetics who have wounds. Both acute and chronic diabetic wounds are characterized by slow, uncoordinated, and partial wound healing (Chen et al., 2020; Li et al., 2024). Impaired and untimely wound healing can result in diabetic foot ulcers (DFU) (Iqbal et al., 2024).

The connection between diabetes and chronic wounds is linked with DFU or diabetic wounds. It is one of the most perilous and recurrent chronic consequences connected with diabetes (Wang H. et al., 2021; Kurkela et al., 2022). The prevalence of DFU in Brazil is 21.0%, whereas Southeast Asia has a range of 10.0%–30.0%, while from 1.0% to 17.0% is found in Europe, and the incidence varies from 5.0% to 20.0% in the Middle East or North Africa (Kalan et al., 2019; Khattak et al., 2025). DFU has a global occurrence rate of 9.1–26.1 million per annum (Mohsin et al., 2024). Approximately 15–25 percent of diabetics encounter DFU, which is an open wound located on the bottom edge of the foot.

Likewise, the process of Wound Healing is intricate and has a brain system (Li et al., 2022a; Yuan et al., 2022). As a result, diabetics who have wounds are more likely to develop chronic involves several steps, including contraction, angiogenesis, fibroplasia, inflammation, coagulation, and tissue remodeling (Baveloni et al., 2025). Also, the complex origins and elevated risk of infection, diabetic wounds are difficult to treat, and these wounds heal more slowly and are more prone to reappear than other types of injuries (Chen et al., 2020; Li et al., 2024).

Furthermore, delayed or nonhealing wounds are among the major consequences associated with hyperglycemia. Many factors can delay healing mechanisms, such as age-related changes in normal physiological function and unfavorable environmental conditions (Patel et al., 2019). There is a possibility that diabetic patients have relatively minor wounds, but these minor wounds may lead to chronic, nonhealing ulcers that are responsible for further contamination and can lead to gangrene and sometimes amputation. The highest amputation rate has been reported in diabetic patients (McDermott et al., 2023). The complex phenomenon of Wound Healing takes place when the anatomic properties of the skin are lost, and the barrier function of the skin is impaired (Spampinato et al., 2020). Subsequently, chronic nonhealing wounds seriously affect the wellbeing and efficiency of patients; hence, they are considered one of the most critical and recurring medical problems in diabetic patients (Shoham et al., 2018). Furthermore, most non-healing wounds exhibit bacterial colonization and biofilm development (Sari et al., 2025). Therefore, antimicrobial therapy is often required for successful wound healing (Rybka et al., 2022). Also, erythema and redness are typical indicators of delayed healing, which can progress to a systemic infection if left untreated. As a result of systemic infection, sepsis may occur, followed by multiorgan failure and eventual death (Price et al., 2021). Because of their complexity and protracted healing phase, diabetic wounds pose a substantial problem in the medical industry. Therefore, there is an urgent need for cutting-edge wound healing treatments that might help diabetic patients heal more quickly and efficiently, encourage tissue regeneration that can enhance Wound Healing.

Medicinal plants are rich in bioactive compounds that act as both antimicrobial agents and free radical scavengers, hence, beneficial for Wound Healing and skin rejuvenation (Thangapazham et al., 2016; Barku, 2018). The strengths of modern scientific techniques can be combined with the development of new functional compounds with increased therapeutic potential to develop plant-based Wound Healing drugs (Barku, 2018).

In this review, we have covered various topics, including the pathophysiology of Wound Healing, factors affecting Wound Healing, some of the phytochemicals that are effective in treating diabetic wounds, their molecular targets, as well as their mechanisms of action. Additionally, this review study will allow researchers to gain a deeper understanding of recent advancements in nanotechnology-based developments for active treatment strategies for diabetic wounds.

## 2 Pathophysiology of diabetic wounds

**FIGURE 1:**
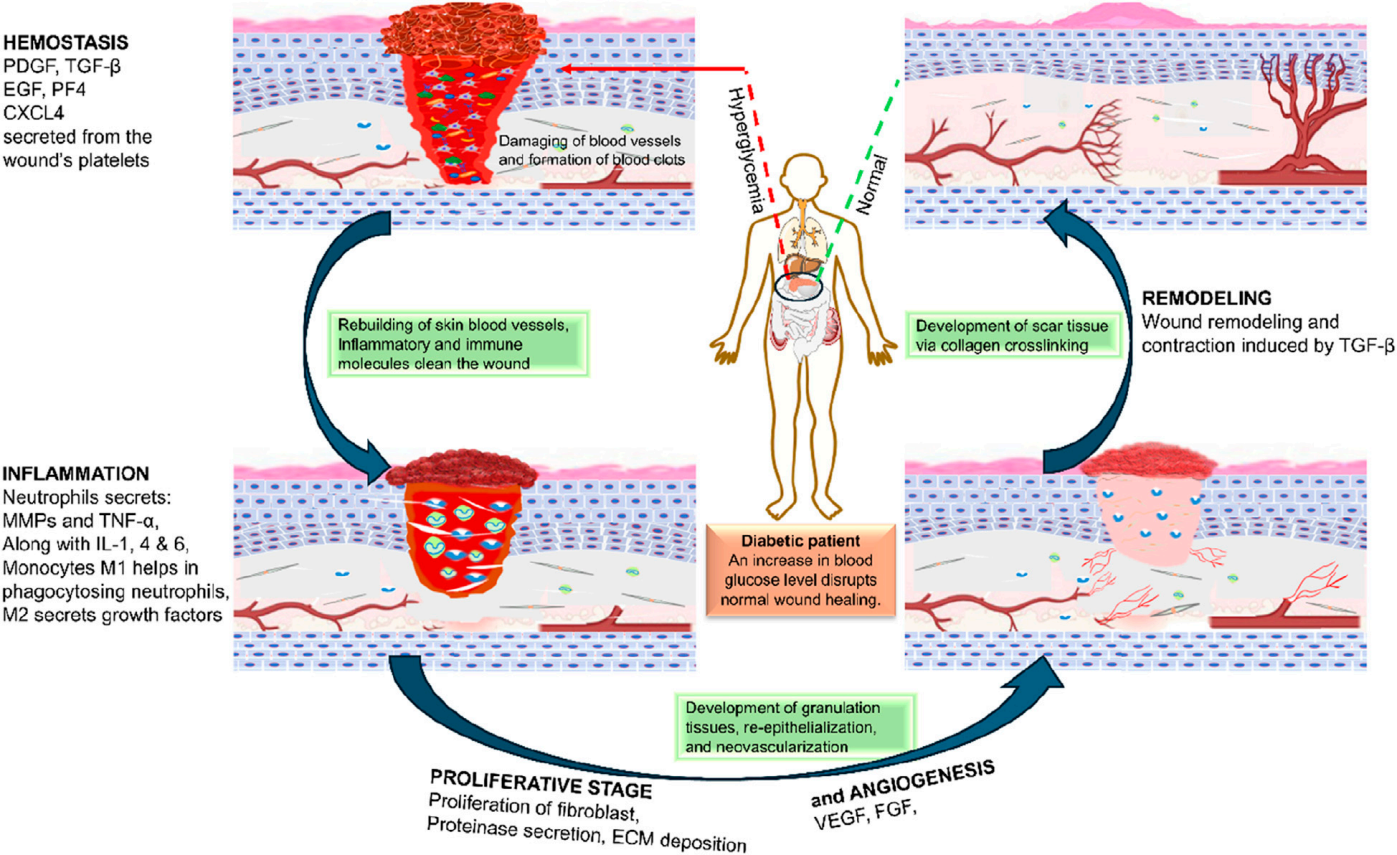
A diabetic patient’s Wound Healing process (PDGF-Platelet-Derived Growth Factor, TGF β- Transforming Growth Factor β, TNF α- Tissue Necrosis Factor, EGF- Epidermal Growth Factor, IL- Interleukins, MMPs-matrix metalloproteases, VEGF- Vascular endothelial growth factor, CXCL4-chemokine (C-X-C motif) ligand 4).

The phenomenon of Wound Healing is divided into four distinct phases. These phases include hemostasis, inflammation, proliferation, and remodeling, as shown in Figure 1.

Hemostasis (Phase I) is a clotting process in which platelets reach the injury site to adhere to collagen type 1 and activate, releasing glycoproteins that cause their aggregation (Oguntibeju, 2019) and resulting in the release of transforming growth factor-β (TGF-β), platelet-derived growth factor (PDGF), endothelial growth factor (EGF), and fibroblast growth factor (FGF) (Ezhilarasu et al., 2020). The interaction of growth factors causes thrombin production, which in turn stimulates fibrinogen formation and the intrinsic coagulation cascade. In addition, when blood vessels are injured, they constrict within minutes, and reducing the level of bleeding can be accomplished by taking various steps that allow hemostasis to occur (Clark, 1994; Oguntibeju, 2019). In case of diabetes, high blood glucose and oxidative stress interfere with platelet aggregation and clotting factors due to hyperglycemia and advanced glycation products, which increase the sensitivity of platelets for spontaneous aggregation, causing rapid consumption and increased probability of reduced availability during hemostasis (Rodriguez and Johnson, 2020) and cause endothelial dysfunction, interfering with hemostasis (Patel et al., 2019). These factors also affect the secretion of TGF-β, PDGF, EGF, and FGF.

Inflammation (Phase II)- Immediately after hemostasis, the inflammation phase starts the movement of inflammatory agents at the injury site (Ezhilarasu et al., 2020). It may take up to 2 weeks or more for the inflammation to subside. Constriction of blood vessels and platelet aggregation, followed by phagocytosis to produce inflammation at the targeted site, are the characteristics of this phase (Oguntibeju, 2019), which occurs due to the release of inflammatory particles such as histamine, prostaglandin, and leukotrienes to promote angiogenesis and cell permeability at the site of the wound (Jonidi Shariatzadeh et al., 2025). One of the major hallmarks of wounds in diabetic patients is the presence of a prolonged inflammatory phase. It often results in delayed healing processes and a risk of chronic wounds. It is caused by the dysregulated function of macrophages and neutrophils as discussed in detail in the next section.

Proliferation (Phase III): The proliferation phase lasts from a few days to a few weeks. This phase is characterized by angiogenesis and the formation of an extracellular matrix (ECM), such as collagens, granulation, and epithelialization (Khatoon et al., 2024). During tissue granulation, fibroblasts form a bed of collagen by transforming into the myofibroblast phenotype, with an augmented alpha-smooth muscle actin (α-SMA) cytoskeleton, which is critical for promoting Wound Healing (van de Water et al., 2013). To reduce the contraction of wound edges, they are pulled together, and new epidermal tissues are formed at the wound site (Fitridge et al., 2024). Delayed angiogenesis is one of the major steps for the development of chronic wounds in case of patients suffering from diabetes. This further limits the supply of nutrients and oxygen to hypoxic tissue (due to the deranged function of microcapillaries because of hyperglycemia), finally leading to delayed tissue regeneration.

Remodeling (Phase IV): The last phase is reported to persist from 3 weeks to 24 months. Throughout the time of remodeling, new collagens are synthesized, along with increased tissue tensile strength (Oguntibeju, 2019). In patients suffering from diabetic complications, as indicated in the next section, there are microvascular complications in the peripheral tissues, and impaired angiogenesis leads to delays in the remodeling phase. Extracellular matrix formation is also hindered due to an altered microenvironment, especially due to dysregulated microphages (Krzyszczyk et al., 2018), which is further complicated by microbial infections. This may lead to ulceration, or, if healing occurs, poor collagen strength in the healed wound, increasing the probability of re-ulceration (Fitridge et al., 2024).

## 3 Complications associated with diabetic wound healing

**FIGURE 2:**
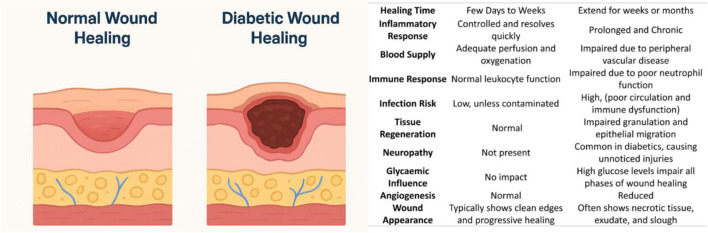
Differential Mechanisms in normal Wound Healing vs. diabetic Wound Healing.

Compared with nondiabetic patients, diabetes mellitus patients are prone to infections and tend to have more severe infections, as shown in Figure 2. There is compelling evidence that diabetic patients are more likely to have certain infections (Holt et al., 2024). Micro- and macrovascular disease and inadequate angiogenesis are likely contributing factors (Singh et al., 2025). Long-term hyperglycemia is strongly associated with microvascular damage, i.e., neuropathy, nephropathy, and retinopathy, which reduce life expectancy, microvascular complications, and other aspects of quality of life (Singh et al., 2025). The clotting process is disrupted in diabetes, and the inflammatory response is sometimes exaggerated and prolonged, resulting in reduced angiogenesis, weakened wound contraction, and decreased wound strength. Diabetic wounds cause hypoxia, which intensifies inflammation. Thus, numerous factors alter the wound-healing process in diabetic patients, resulting in several complications (Fitridge et al., 2024) Figure 3.

**FIGURE 3:**
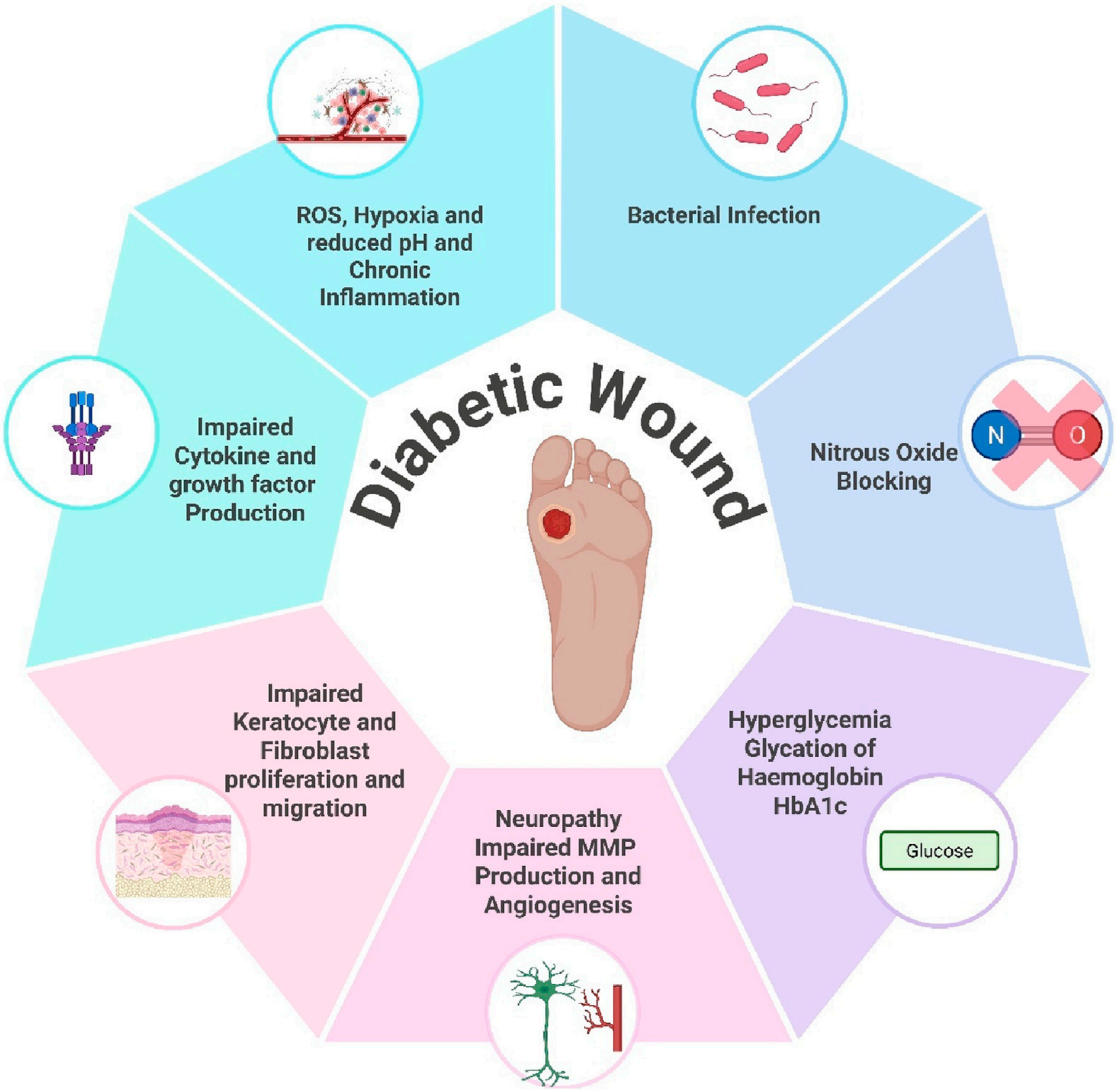
Major factors affecting wound healing in diabetic patients (made using biorender.com).

### 3.1 Poor blood circulation and oxidative stress

Higher blood glucose levels cause the blood to thicken, which impairs blood circulation and makes it harder for blood cells to move through the body. As a result, the body parts do not receive an adequate blood supply, resulting in oxygen and nutrient deficiency, which impairs cellular metabolism and delays the healing process (Chen et al., 2016).

Additionally, the development of sugar-derived highly oxidant substances can obstruct the healing process of wounds in diabetic patients by inhibiting inflammation and proliferation. These compounds are called advanced glycation end products (AGEs) or glycotoxins (Kang et al., 2021). Free sugars are frequently very reactive and are subject to non-enzymatic degradation under physiological conditions, which leads to the generation of intermediary compounds that can react with other molecules, potentially causing harmful effects. This group of intermediary and advanced products is referred to as glycotoxins (Monteiro-Alfredo and Matafome, 2022). It is a diverse category of compounds that include AGEs and their precursors, most of which are highly reactive intermediary compounds.

Furthermore, free radicals are generated by AGEs, resulting in an imbalance between free radicals and antioxidants in the body, which causes oxidative stress, tissue damage, and slow Wound Healing. When free radicals react with proinflammatory cytokines, they promote the production of serine proteinases and matrix metalloproteinases that damage and disable components of the extracellular matrix (ECM) and growth factors required for normal cell function (Fitridge et al., 2024).

### 3.2 Impaired immune response

Cellular dysfunctions such as defective and impaired T lymphocytes, chemotaxis, phagocytosis, and bactericidal capacity also play important roles in the delayed Wound Healing of diabetic patients (Houreld, 2014). The Wound Healing rate becomes sluggish because of compromised immune function, resulting in an increased risk of infection. When such infections are left untreated, many severe complications, including sepsis and gangrene, can occur. Abnormally extended activation and dysregulated apoptosis of neutrophils in a high-glucose wound microenvironment lead to an abnormally high release of neutrophil extracellular traps (NETs), resulting in NETosis. This phenomenon ultimately culminates in reduced angiogenesis and prolonged inflammation in the wound (Xiao et al., 2024). Changes in Macrophage phenotypes in high glucose and oxidative microenvironment lead to exclusive proinflammatory M1 phenotype, promoting inflammation and delaying wound healing in the case of diabetes. Therapeutic approaches promoting conversion of M1 macrophages to an Anti-inflammatory M2 phenotype or promotion of recruitment of M2 macrophages to the wound microenvironment can lead to the resolution of inflammation and hastened healing (Fu et al., 2020; Wu et al., 2022). Although Mast cells play a role in wound healing but their role in impaired diabetic wounds is not well defined. In diabetic wounds, infiltrating cells release elevated levels of inflammatory cytokines, such as tumor necrosis factor-alpha (TNF-α), interleukin-6 (IL-6), and interleukin-1β (IL-1β), which persist at high concentrations for extended periods, thereby prolonging the inflammatory phase (Oyebode et al., 2023).

### 3.3 Neuropathy

Diabetic complications damage the nerve, reducing the ability to feel or perceive wounds and their pain (neuropathy) in the limbs, feet, or other parts of the body. Consequently, diabetic patients who have cuts, burns, and blisters that are untreated are more likely to become seriously infected, and their wounds heal more slowly. Moreover, individuals with diabetes are prone to small problems that may become major problems if they are not detected. Neuropathy is common in the hands and feet (Feldman et al., 2019). In addition, dysregulation of secretion of neuropeptides from the nerves, especially substance P and Calcitonin gene-related peptide, has effects on vasodilation and migration of cells to the wound tissue as well as enhances the expression of NO, a potent vasodilator. Substance P is also involved in the activation of macrophages and the secretion of cytokines. The detailed role of all neuropeptides in wound healing is discussed in Table 1.

**TABLE 1:** Effect of Neuropeptides secreted from Neurons on wound healing ((Xing et al., 2024)).

Neuropeptide	Inflammatory phase	Proliferative phase	Remodeling phase
Substance P (SP)	Activates macrophages	Stimulates fibroblast proliferation and migration	Modulates MMP expression and activity
	Promotes cytokine release	↑ collagen synthesis and ECM formation	Regulates TGFβ signaling
	Recruits and activates immune cells via NK1R	↑ Wound strength and structural integrity	Facilitates ECM remodeling
Calcitonin Gene Related Peptide (CGRP)	Interacts with neutrophils and macrophages via RAMP1	Promotes fibroblast proliferation and migration	Stimulates angiogenesis via TSP1
	Inhibits immune cell recruitment	↑ Collagen I expression	Reduces immune cell infiltration
	Promotes immune cell apoptosis		Enhances immune cell apoptosis
Neuropeptide Y (NPY)	Enhances TNFα production via Y1R	Regulates TNFα via βarrestin 2	Promote collagen fiber crosslinking and alignment
	Inhibits IL1β release	Supports angiogenesis and oxygen/nutrient supply	Facilitates wound contraction
	Limits excessive inflammation		Influences αactin expression for tissue remodeling
Vasoactive Intestinal Peptide (VIP)	Inhibits NO, ILs, and TNFα release	Stimulates cell growth and differentiation via VIPRs	Regulates cAMP signaling
	↑ IL10 synthesis	Accelerates tissue repair and regeneration	Modulates immune cell function
	Prevents T cell overactivation		Supports tissue remodeling

### 3.4 Malformation of the ECM

The formation of the extracellular matrix (ECM), an important healing mediator, contributes to the tissue being structurally stable (Houreld, 2014), facilitates transduction of the signal and coordinates cell‒cell and cell‒matrix interactions. In diabetic wounds, the ECM is distorted due to the disturbed interaction between the ECM and growth factors (such as TGF-β and VEGF), which are necessary for normal cell function. This, in turn, results in impaired proliferation, migration, and differentiation of fibroblasts, and there is an imbalance between matrix-degrading enzymes, MMPs, and their inhibitors, and tissue inhibitor metalloproteinases. The balance between matrix synthesis and degradation is essential for collagen synthesis and maintenance in ECM. Therefore, diabetes results in decreased collagen production and increased metabolism, or sometimes a combination of both (Tombulturk et al., 2024).

Therefore, it is now understood that the delayed or impaired healing of wounds that occur in patients with diabetes is caused by both molecular and cellular abnormalities involving a reduction in the host’s immune response, neuropathy, damage from reactive oxygen species, and advanced glycation end products, abnormalities in fibroblast and epidermal cell functions, hypoxia, impaired angiogenesis, and elevated levels of MMPs. Nevertheless, it remains a challenge for healthcare professionals to make an accurate diagnosis, select effective treatments, and prevent wound recurrence.

### 3.5 Wound microbiota

Nearly 50% of diabetic wounds are complicated by the presence of a diverse microbial population (Prompers et al., 2007). Chronic wounds are generally inhabited by microorganisms of the following species, i.e., *Staphylococcus* spp., *Pseudomonas* spp., *Corynebacterium* spp., *Enterococcus* spp., *Streptococcus* spp., and *Cutibacterium* spp. With increased severity. Cells present in the wound and adjacent tissues undergo programmed cell death through various mechanisms induced by high oxidative stress. These processes lead to the conversion of chronic wounds into necrotic wounds. Wound management necessitates extreme measures such as amputation in the worst cases, to save the patient. This condition is further complicated by the presence of various bacteria and fungi, most notably biofilm-forming bacteria such as *S. aureus* (MRSA) and *P. aeruginosa* (Pranantyo et al., 2024). These biofilms protect bacteria from the host immune system as well as antibiotic therapy thereby prolonging their residence on wounds and development of antibiotic resistance (Razdan et al., 2022). This condition necessitates the use and development of topical therapies able to target a matrix of inflammation, oxidative stress, biofilms, and microbial contaminations.

### 3.6 Clinical approaches for the treatment of diabetic wounds

The clinical management of diabetic wounds utilizes a multifaceted approach that involves a combination of USFDA-approved therapies, adjunctive therapies, and some experimental approaches. The therapy is initiated with wound assessment and proceeds with key aspects such as offloading pressure from the wound, debridement, infection prevention/control and glycemic control. Topical treatment, such as ointments and dressings, plays a very important role in controlling local infection and promoting wound healing.

Treatment of diabetic wounds includes growth factors, acellular matrix, bioengineered tissue, negative-pressure therapy, stem cell therapy, and topical and hyperbaric oxygen therapy (Elsayed et al., 2023). Many of these treatment options are not backed up by robust clinical trials. FDA-approved therapies include Becaplermin, containing platelet-derived growth factor, PDGF, which was one of the first therapies to be approved by the USFDA for Diabetic foot ulcer. In addition to that, Apigraf and Dermagraft have also been approved as a wound dressing for Diabetic foot ulcers lasting more than 4 weeks. The major disadvantage of these dressings cannot be used for wounds with infections (Barakat et al., 2021). Integra’s Omnigraft Dermal Regeneration Matrix, originally approved for burns, also got approval for DFU. One of the recent additions to the list is SkinTE, an organoid-like culture obtained from harvested skin for the therapy of Wagner grade 1 diabetic foot ulcer (Armstrong et al., 2023).

Antibiotics are also a mainstay for therapy in the presence of microbial infections. Apart from parental and oral administration of antibiotics, topical antibiotics have also been used for the therapy of microbial infection in diabetic wounds. Recently, the USFDA has given Pravibismane approval for the topical therapy of diabetic wounds contaminated with microbial infections (Lipsky et al., 2024). The drug is different from current microbial therapy due to its novel structure and effectiveness against biofilm formation.

## 4 Phytoconstituents used for the topical therapy of diabetic wounds

**FIGURE 4:**
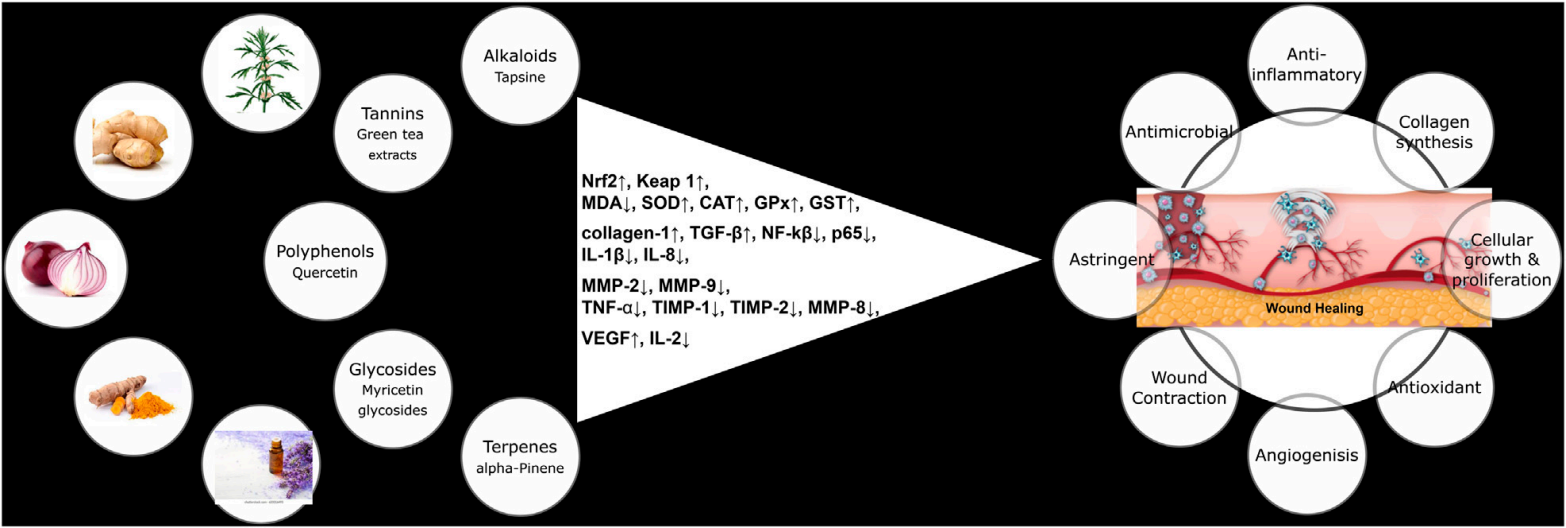
Different phytochemicals, their molecular targets, and pharmacological effects in the context of diabetic wound healing.

Medicinal plants contain various phytoconstituents, including micronutrients, amino acids, proteins, resins, mucilage’s, essential oils, terpenes, and/or triterpenoids, sterols, saponins, carotenoids, alkaloids, flavonoids, tannins, and phenolic acids, which have significant roles in therapeutic activity (Teoh and Das, 2018). In recent years, increasing evidence has shown that phytochemicals can be useful in enhancing Wound Healing both acutely and chronically (Shah and Amini-Nik, 2017). Topical applications of natural medicinal products and plant extracts have long been utilized for wound management. The discovery and preclinical studies of phytochemicals suggest that phytoconstituents could be beneficial for Wound Healing, skin regeneration, and may prevent and/or treat a variety of other deadly diseases (Karma et al., 2025). The mechanism of phytochemical-mediated improved diabetic Wound Healing includes antimicrobial, antioxidant, debridement, and anti-inflammatory effects, and may provide a hydrated environment. Figure 4 represents the various phytoconstituents and their probable molecular targets. The majority of Wound Healing pharmaceutical products are plant-based; only 20% are mineral-based, and 10% are animal-based (Ahmed et al., 2018). Various natural compounds that can have wound-healing properties are discussed below.

### 4.1 Flavonoids and polyphenols

Several bioactive compounds found in plants are known to accelerate healing. Flavonoids are among the most important bioactive constituents in plants (Ahmad et al., 2017). Flavonoids have a polyphenolic structure (Ullah et al., 2020) and are well known for their ability to modulate the body’s response to various diseases (Ahmad et al., 2017). They are also responsible for a wide range of pharmacological effects (Ullah et al., 2020). According to research, quercetin stands out as the most potent antioxidant among the experimental bioflavonoids. Many foods contain quercetin, including apples, berries, nuts, red wine, Chinese herbs, and vegetables such as cauliflower, cabbage, and onions. The antioxidant properties of quercetin contribute to the prevention of diabetes and related complications such as diabetic nephropathy, cardiovascular disorders, delayed wound healing (Yan et al., 2022), retinopathy, and diabetic neuropathy, as demonstrated in numerous recent studies (Salehi et al., 2020). Several factors contribute to the hypoglycemic activity of polyphenols, including lowering the intestinal assimilation of dietary carbohydrates, regulating glucose-metabolizing enzymes, and stimulating insulin production (Rocha et al., 2025). Quercetin reduces intestinal glucose absorption and enhances glucose uptake in organs and tissues by increasing the expression of silent information regulator 1 (SIRT1) and peroxisome proliferator-activated receptor-γ (PPARγ). This leads to the activation of 5′adenosine monophosphate-activated protein kinase (AMPK) and improves insulin resistance, thereby reducing blood glucose levels. Oxidative stress can worsen diabetes and its complications by impairing insulin secretion and increasing insulin resistance. As a powerful antioxidant, quercetin slows the progression of diabetic complications by preventing oxidative stress (Shi et al., 2019), thereby protecting the β-cells of the pancreas (Vessal et al., 2003). Mast cells, neutrophils, and macrophages contribute to inflammation through the release of chemokines, cytokines, and free radicals. This immune activity causes inflammation in pancreatic islets and promotes peripheral insulin resistance (Shi et al., 2019). Quercetin may therefore be effective in treating these conditions by suppressing the release of inflammatory factors such as TNF-α and by blocking TNF-α–mediated inflammation (Li et al., 2016). It has antifibrotic [85], antibacterial (Qi et al., 2022; Alishahi et al., 2024; Almuhanna et al., 2024), anti-inflammatory (Lee et al., 2024), anti-atherosclerotic (Wang Y. et al., 2024; Xiang et al., 2024), anticarcinogenic (Hasan et al., 2025), Wound Healing, and diabetic Wound Healing properties (Li et al., 2022a; Almuhanna et al., 2024; Huang et al., 2024; Yadav et al., 2025). Quercetin significantly increased wound contraction through enhanced epithelialization, possibly due to its ability to elevate tissue antioxidant levels. Its antifibrotic and antihistaminic properties could make it an effective treatment for hypertrophic scars (Ahmad et al., 2017). Therefore, quercetin can improve common wound healing by increasing fibroblast proliferation while decreasing fibrosis and scar formation (Jangde et al., 2018). By suppressing inflammation, promoting collagen deposition, aiding fibroblast proliferation, speeding angiogenesis, and regulating oxidative stress, quercetin has been shown to have a promising effect on Wound Healing (Huang et al., 2024). Quercetin also averts endogenous antioxidant depletion, guards keratinocytes from free radicals, hinders UV-induced lipid peroxidation, and reduces the release of pro-inflammatory cytokines (Hatahet et al., 2016; Chappidi et al., 2024).

Turmeric contains bioactive compounds known as curcuminoids, which are responsible for its yellow color. Curcumin, desmethoxycurcumin, and bisdemethoxycurcumin are the three major curcuminoids found in turmeric. Among all the active compounds, curcumin is the most abundant and biologically active secondary metabolite (Quispe et al., 2022). It is a crystalline compound with an orange‒yellow hue (Akbik et al., 2014), lipophilic polyphenolic nature (Hussain et al., 2022), and practically insoluble in water (Akbik et al., 2014). Curcumin possesses anti-tumor, anti-aging, and antioxidant properties (Abdul-Rahman et al., 2024), anti-inflammatory (Abdul-Rahman et al., 2024), antihyperglycemic (Mohammadi et al., 2021), immuno-modulating (Memarzia et al., 2021), anti-anxiety (Fathi et al., 2024) neuroprotective (Genchi et al., 2024; Khayatan et al., 2024), antidepressant (Ng et al., 2017) and wound-healing properties (Adamczak et al., 2020; Jakubczyk et al., 2020; Fan et al., 2024; Wang X. et al., 2025). For centuries, inflammation and wounds have been treated with curcumin paste mixed with lime (Akbik et al., 2014). In addition to its anti-inflammatory effects, curcumin inhibits nuclear factor kappa-B and may help prevent and manage diabetes (Maradana et al., 2013). Various mechanisms contribute to curcumin’s ability to ameliorate diabetic pathologies, including lipid metabolism regulation and antioxidant activity. Curcumin has been shown to mitigate diabetic complications both directly and indirectly, including neuropathy, nephropathy, retinopathy, atherosclerosis, and delayed wound healing (Quispe et al., 2022).

Curcumin promotes wound healing by modulating inflammation through the inhibition of the cytokines TNF-α and IL-1 (Dehghani et al., 2020). Furthermore, several biological mechanisms contribute to curcumin’s regenerative effects on diabetic wounds, including angiogenesis, reduced oxidative stress, increased cell proliferation, and enhanced collagen production (Li et al., 2022b). Studies have concluded that wounds treated with curcumin exhibit increased re-epithelialization, enhanced neovascularization, and higher collagen content compared to those treated with conventional drugs.

Effective wound healing can be achieved through oral administration, topical application, or a combination of both. However, curcumin’s therapeutic application is limited due to poor bioavailability, rapid metabolism, and a short half-life. Its hydrophobic nature and low aqueous solubility result in poor absorption, which constrains its topical efficacy. Furthermore, the polyphenolic nature of curcumin can sometimes lead to toxic effects when applied topically at high concentrations. Currently, various efficient delivery systems have been developed to optimize the therapeutic utility of curcumin in topical therapy, aiming to improve its solubility, prevent hydrolysis, and ensure sustained release (Mohanty and Sahoo, 2017). Limitations of curcumin has been successfully addressed using nanotechnology via development of different nanocarriers.

Epigallocatechin-3-gallate (EGCG), a polyphenol found in green tea, has garnered considerable interest in recent years due to its potent antioxidant activity, which contributes to the treatment of cutaneous wounds by promoting re-epithelialization. It also exhibits bactericidal and anti-inflammatory effects and facilitates angiogenesis (Zhao et al., 2021). It inhibits the signaling cascades of platelet-derived growth factor (PDGF) and epidermal growth factor (EGF) during the inflammatory phase. Mast cells, neutrophils, and macrophages contribute to inflammation through the release of free radicals, cytokines, and growth factors. EGCG suppresses the platelet-derived growth factor receptor while enhancing microvascular blood flow. By suppressing interleukin-8 production, EGCG reduces neutrophil aggregation, thereby inhibiting the inflammatory response and modifying the nitric oxide synthase pathway, which decreases both inflammation and reactive oxygen species production.

Furthermore, EGCG stimulates the cleavage of enzymes that accelerate wound healing by eliminating free radicals. By inhibiting nitric oxide production, EGCG serves as an antioxidant because of its ability to neutralize free radicals and maintain wound healing. Endothelial cells in the vascular system are also effectively protected by EGCG (Zawani and Fauzi, 2021). Despite its therapeutic potential, EGCG suffers from poor bioavailability and rapid metabolism, which limit its clinical utility. Therefore, the development of advanced drug delivery systems that release EGCG in a controlled and targeted manner is highly desirable [85]. Consequently, nanoscale formulations have been extensively explored to enhance the stability and therapeutic efficacy of EGCG (Sun M. et al., 2020). Kaempferol, also known as 5,7-trihydroxy-2 (4-hydroxyphenyl)-4H-1-benzopyran-4-one, is a flavonol found in many plants, fruits, and vegetables. It possesses a wide range of pharmacological properties, including antioxidant, anti-inflammatory, anticancer, cardioprotective, and antidiabetic activities. Kaempferol and its glycosides exhibit antioxidant properties, effectively scavenging peroxynitrite ions, hydroxyl radicals, and superoxides. Additionally, it inhibits xanthine oxidase and other ROS-generating enzymes and is well known for its ability to reduce intracellular ROS accumulation. Zeng et al. confirmed that kaempferol hindered the formation of neutrophil extracellular traps (NETs) by reducing ROS production from NADPH oxidase (Zeng et al., 2020). In HUVEC cells, kaempferol was shown to activate the Nrf2 signaling pathway, enhance glutathione and catalase activity, and reduce malondialdehyde levels (Yao et al., 2020). Furthermore, kaempferol reduced macrophage-mediated inflammation by decreasing the secretion of pro-inflammatory cytokines and promoting macrophage repolarization through inhibition of ROS-associated NF-κB signaling (Zhao et al., 2023). In diabetic rats, Özay et al. demonstrated that kaempferol promoted wound contraction and re-epithelialization while increasing collagen and hydroxyproline levels (Özay et al., 2019). The antioxidant and anti-inflammatory potential of kaempferol likely underlies its effectiveness in accelerating wound healing in diabetic patients (Özay et al., 2019). The antioxidant and anti-inflammatory potential of kaempferol can be responsible for its effectiveness in delaying wound healing in diabetic patients.

Luteolin (a 3,4,5,7-tetrahydroxy flavone) is present in many fruits, plants, and vegetables. It has many biological effects, including anti-allergic, anti-inflammatory, and anti-cancer properties, along with pro-oxidant or antioxidant activities (Ambasta et al., 2019). Chen et al. have confirmed the capability of luteolin to recover Wound Healing in diabetic rats by accelerating collagen deposition and re-epithelialization (Chen et al., 2021). Furthermore, luteolin decreases the formation and accumulation of terminal glycation products (AGEs) in the HSA (human serum albumin)/glyoxal system (Sarmah et al., 2020). Its anti-inflammatory action can be elucidated by its ability to modulate macrophage polarization. Indeed, Wang et al. observed LPS-activated RAW264.7 cells in the presence of luteolin decreased the M1 polarization surface markers and increased the M2 surface markers (Wang et al., 2020), which aligns with their cytokine secretions. Luteolin can also impede NLRP3 inflammasome activation and IL-1β secretion in J774A.1 macrophage by modulating ASC oligomerization. Hence, it can be presumed that H2O2 scavenging by luteolin or the diminution in intracellular ROS might reduce the activation of the NLRP3 inflammasome and reduce the M1 polarization of macrophages to recover Wound Healing (Wang et al., 2020).

### 4.2 Berberine

Berberine (BBR) is a quaternary benzylisoquinoline alkaloid obtained from the genus *Berberis* of Berberidaceae family. Several medicinal plant species, including *Argemone mexicana, Coscinium fenestratum, and Tinospora cordifolia,* contain berberine in their bark, leaves, twigs, roots, and stems (Neag et al., 2018). Berberine extracts are well known for their anthelminthic, antibacterial, antiviral, antifungal, and antiprotozoal effects (Neag et al., 2018). It is also reported to increase the activity of antioxidant enzymes (e.g., GPx, SOD) thereby suppressing inflammation and oxidative stress (Amato et al., 2021). Many studies have shown that berberine stimulates multiple metabolic processes. It may prevent hyperglycemia, cardiovascular diseases such as hyperlipidemia and hypertension, cytotoxicity, and inhibitory effects on the growth and reproduction of certain tumorigenic organisms, depression, and inflammatory diseases (Pang et al., 2015). Berberine heals wounds via various mechanisms, including the activation of silent information regulator 1 (Sirt1) (Qiang et al., 2017), which is a nicotinamide adenine dinucleotide-dependent type III histone deacetylase (Zhang et al., 2020) and plays a crucial role in the body’s inflammation, immune system response and, ultimately, wound repair; moreover, it increases the expression of vascular endothelial growth factor, which is a potent angiogenic factor, whereas tumor necrosis factor-α, interleukin-6, and nuclear factor kappa B expression are inhibited, which benefits the healing of diabetic wounds (Qiang et al., 2017; Panda et al., 2021).

Reports have shown that with BBR treatment, Wound Healing is remarkably accelerated, extracellular matrix synthesis is enhanced, and the damage caused by high glucose to culture the human keratinocytes (HaCaT) is significantly inhibited. Further studies suggested that berberine activates thioredoxin reductase-1 (TrxR1) and inhibits c-Jun N-terminal kinase signaling, thus accelerating Wound Healing by inhibiting apoptosis and oxidative stress, promoting the proliferation of cells, and upregulating transforming growth factor-β1 (TGF-β1), which downregulates matrix metalloproteinase-9 (MMP-9) and tissue inhibitors of metalloproteinase-1. The results indicate that topical berberine treatment can enhance wound closure in diabetic patients (Zhou et al., 2021).

### 4.3 Paeoniflorin

Paeoniflorin (PF) is a monoterpene glycoside obtained from the dried and peeled roots of *Paeoniae lactiflora* pall, belonging to the family *Paeoniaceae*. It possesses many pharmacological and biological functions. PF is widely known for its anti-inflammatory and antioxidant properties (Sun et al., 2021). Menstrual cramps and abdominal spasms are both treated with paeoniflorin as a pain-relieving medication. According to a previous study, it also protects against oxidative injuries induced by high glucose levels in cells by triggering the nuclear factor erythroid 2–related factor 2/antioxidant response element signaling (Nrf2/ARE) pathway and impeding apoptosis. Thus, paeoniflorin treatment can improve the healing of diabetic ulcers through increased nuclear factor erythroid 2–related factor 2 expression. Furthermore, PF inhibits the inflammatory response mediated by cytokines and chemokines (TNF-α, IL-1β, and monocyte chemoattractant protein-1) by inhibiting Toll-like receptor 2/4 (TLRs 2 and 4) and reduces related complications, such as nephropathy, in diabetic patients.

In Wound Healing, nuclear factor erythroid 2–related factor 2 has been identified as a good target, regardless of whether a patient is diabetic. Nuclear factor erythroid 2–related factor 2 is involved in corneal epithelial Wound Healing through its ability to promote cell migration. Another study revealed that increased nuclear factor erythroid 2–related factor 2 levels reduce oxidative stress and promote swift Wound Healing in diabetic mice. Therefore, as a result of activating nuclear factor erythroid 2–related factor 2, PF accelerates healing, reduces oxidative stress, increases cell proliferation, and decreases apoptosis. Diabetic wound tissues that had been treated with PF presented significantly increased levels of vascular endothelial growth factor and transforming growth factor-β1. The expression of C-X-C motif chemokine receptor 2 (CXCR2) and nuclear factor kappa light chain enhancer in activated B cells was also decreased by paeoniflorin, and that of IκB was increased. Enhanced wound contraction was also observed in the paeoniflorin-treated group because of decreased Nod-like receptor protein-3 (NLRP3) and caspase-1 levels. Through the inhibition of C-X-C motif chemokine receptor 2, paeoniflorin effectively mitigated nuclear factor kappa B- and Nod-like receptor protein-3-mediated inflammation. The antioxidant and anti-inflammatory properties of paeoniflorin help protect the body from vascular injury induced by fluctuating hyperglycemia. In addition to preventing oxidative stress induced by high glucose, paeoniflorin also suppressed apoptosis by activating the Nrf2/ARE pathway. Through the downregulation of C-X-C motif chemokine receptor 2, paeoniflorin inhibits Nod-like receptor protein-3 inflammasome- and NF-κB-mediated inflammatory reactions in diabetic wounds (the activation of the Nod-like receptor protein-3 inflammasome leads to the maintenance of inflammation and delays Wound Healing). Treatment causes a greater reduction in the levels of proinflammatory cytokines, such as IL-1β, IL-18 and TNF-α, which are highly expressed in the wounds of diabetic patients. By blocking C-X-C motif chemokine receptor 2, paeoniflorin reduces the activation of (Nod-like receptor protein-3) NLPR3/ASC (apoptosis-associated speck-like protein containing a CARD) inflammasomes. NLRP3- and NF-B-mediated inflammatory reactions may be inhibited by paeoniflorin through the blockade of C-X-C motif chemokine receptor 2, which is a possible mechanism of action in diabetic ulcers. The inactivation of TLR4/NF-κB by paeoniflorin has recently been shown to be beneficial in the treatment of diabetic retinopathy (Zhang et al., 2017; Sun X. et al., 2020).

### 4.4 D-pinitol

**FIGURE 5:**
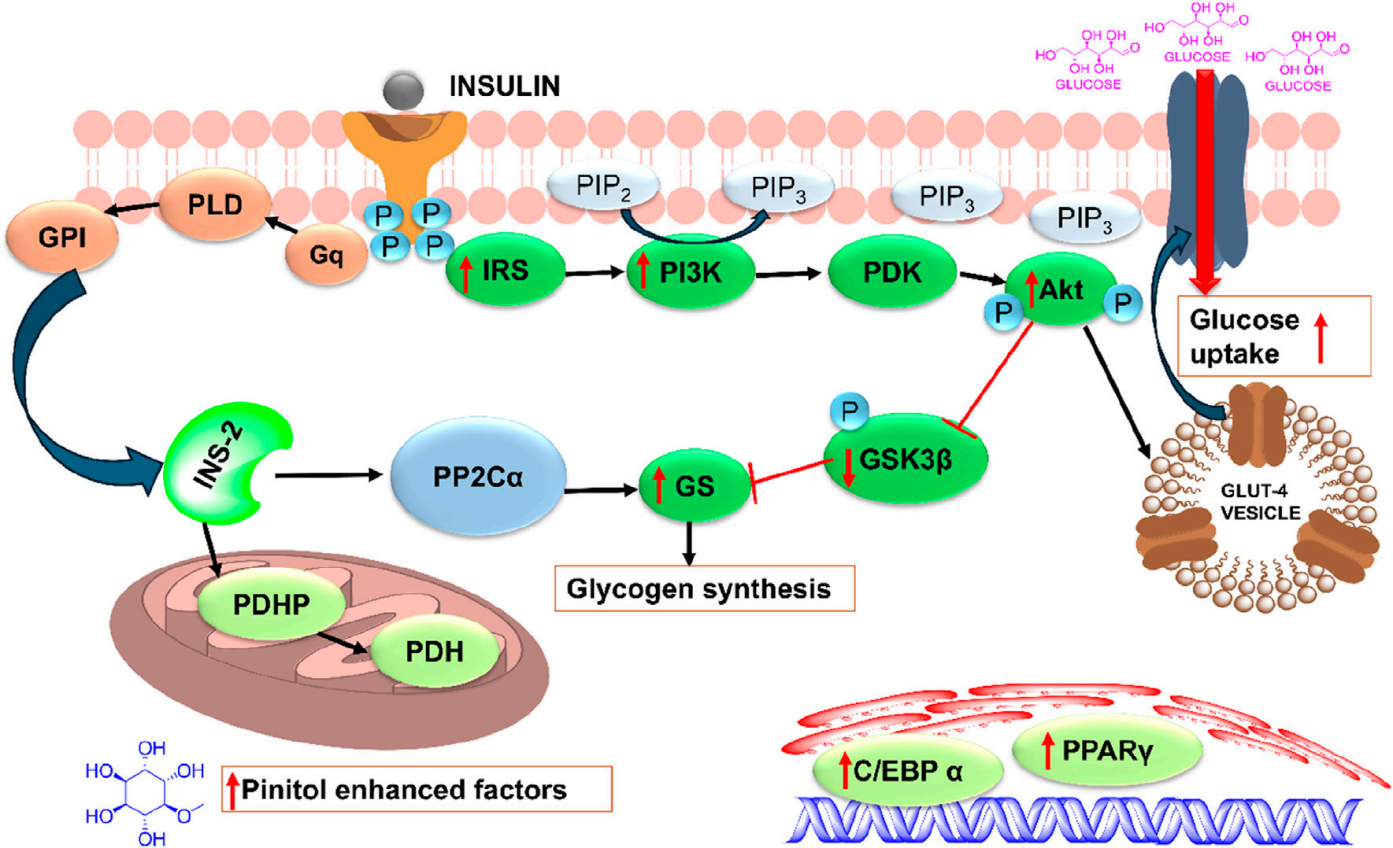
Plausible mechanism of action of pinitol as an insulin sensitizer (Sánchez-Hidalgo et al., 2021). C/EBPα, CCAAT/enhancer binding protein α; GLUT-4, glucose transporter 4; GPI, glycosyl phosphatidylinositol; GSK-3β, glycogen synthase kinase 3β; IR, insulin receptor; INS-2, insulin second messenger; PDH, pyruvate dehydrogenase; PDHP, pyruvate dehydrogenase phosphatase; PI3K, phosphoinositide 3 kinase; PDK-1, phosphoinositide-dependent kinase 1; PP2Cα, phosphoprotein phosphatase 2C α; PKB/Akt, protein kinase B/Akt; PPARγ, peroxisome proliferator-activated receptor γ; PLD, phospholipase D.

D-pinitol is a methyl ether of D-chiro-inositol and is getting attention because of its existence in medicinal plants and foods, including soybean (*Glycine max*), ice plant (*Mesembryanthemum crystallinum*), carob pod (*Ceratonia siliqua*), fenugreek seed (*Trigonella foenumgraecum*), and some species of the Retama genus (Lee et al., 2014; Tetik and Yüksel, 2014; González-Mauraza et al., 2016; Lahuta et al., 2018; Christou et al., 2019; Zhang et al., 2019). Moreover, D-pinitol has been indicated to have multifunctional medicinal properties such as anti-diabetic (Gao et al., 2015), anti-inflammatory (Zheng et al., 2017), antioxidant (Lee et al., 2019), chemopreventive (Rengarajan et al., 2012), antitumoral (Lin et al., 2013) and diabetic foot ulcers (Kim et al., 2024). One reviewed research recommended that D-pinitol may help to enhance insulin resistance and slow the progression of type II diabetes, and they suggested a mechanism of action of D-pinitol as an insulin sensitizer (Figure 5) (Sánchez-Hidalgo et al., 2021).

Kim and colleagues conducted a study on the wound-healing properties of pinitol in lipopolysaccharide-induced human dermal fibroblasts and streptozotocin-induced diabetic rat models with foot wounds and found that pinitol reduced oxidative stress by enhancing the nuclear translocation of Nrf2 and markedly upregulating antioxidant enzymes like HO-1, SOD1, SOD2, and catalase (Kim et al., 2024). Further, pinitol-induced Nrf2 overexpression suppressed the IκBα/NF-κB signaling pathway and reduced inflammatory cytokines such IL-6, IL-1β, and IL-8. Pinitol also improved collagen deposition, reduced matrix metalloproteinase (MMP) activity, and restored mitochondrial energy metabolism. These results suggested that in wound infection models, pinitol-mediated Nrf2 overexpression enhanced wound-healing effects.

### 4.5 Carotenoids

Astaxanthin is a dark reddish carotenoid obtained from *Haematococcus pluvialis* has been very widely used food supplement and is known for its potent antioxidant activity. It has been reported to correct metabolic dysregulation in different diseases, such as diabetes and age-related disorders, and is used as an oral supplement in these disorders (Wang F. et al., 2025). Poor solubility and stability are major hurdles for its development as a therapeutic option for use in diabetic wounds (Zhan et al., 2025). Recently, astaxanthin has been successfully used for diabetic wound healing by loading it into a bilayer nanofibrous membrane made of chitosan and polyvinyl alcohol, enriched with MXene and ZnO nanoparticles, respectively. The membrane, produced using coaxial electrospinning, demonstrated enhanced wound healing by scavenging ROS and reducing inflammation (Zhan et al., 2025). In another study, Astaxanthin was loaded in a nanoemulsion composed of α-tocopherol stabilized with κ-carrageenan. Transdermal administration of nanoemulsions is reported to enhance glycemic control and enhance wound healing and closure by reducing oxidative stress in the wounds (Shanmugapriya et al., 2019). In another study, astaxanthin was stabilized by loading it into chitosan-coated nanocarriers, which were further incorporated into Carbopol gel to enhance wound healing. This effect was mediated by enhanced neovascularization, accelerated epithelialization and collagen deposition, and reduced infiltration of inflammatory cells in the wound (Barari et al., 2025).

### 4.6 New phytochemicals and extracts for diabetic wound healing

*Centella asiatica* extract-loaded hydrogel, which can be easily printed on various surfaces, has been reported to be effective in treating chronic wounds, as the hydrogel ensures complete contact with the wound tissue and reduces inflammation and oxidative stress (Wang X. et al., 2024). In another study, ethanolic flaxseed extract-loaded nanofibrous scaffolds demonstrated enhanced wound closure, along with antimicrobial effects against a wide spectrum of bacteria (Abdelaziz et al., 2025). Self-assembled hydrogels composed of poorly soluble asiaticoside and saponins from *Panax ginseng* were able to enhance wound healing mediated by reduction of IL-6 and enhancing production of VEGF at the wound site, leading to improve collagen fiber organization (Huang H. et al., 2025). Hao et al. have reported a self-assembled hydrogel composed of mangiferin for diabetic wound healing, mediated by the reduction of intracellular ROS, modulation of inflammation, and enhancement of collagen deposition and angiogenesis to promote wound contraction and healing (Hao et al., 2024).

## 5 Current nanotherapeutic approaches for diabetic wound healing

The complications associated with impaired chronic wounds require approaches to decrease the infection rate, accelerate wound closure, reduce scar impressions, and overcome the limitations of existing wound therapies (Vijayakumar et al., 2019). The biocompatibility of phytochemicals makes them good candidates for therapeutic negotiators. However, they have several limitations, including poor biopharmaceutical and pharmacokinetic properties, such as poor solubility in water, rapid metabolism, and short half-lives, which limit their clinical value.

Hence, the nanomaterials employed to improve clinical efficacy with plant-based therapeutic agents have proven to be the most effective approach to overcome pharmacokinetic and biopharmaceutical hindrances (Dewanjee et al., 2020). Nanotechnology-based nanomaterials are a unique and diverse approach that utilizes materials with sizes ranging from 1 to 100 nm to enhance wound repair and reduce complications (Hamdan et al., 2017). Additionally, nanostructures have incomparable properties because of their high surface area-to-volume ratios, and some of them have antibacterial properties (Wang et al., 2022). NPs assembled or loaded with phytoconstituents promote functionalization and reduce the probability of side effects (Dewanjee et al., 2020). A nanoscale particle, for example, enhances penetration into the wound site more effectively and allows it to interact with the biological target. Thus, nanoparticles can deliver therapeutics in a sustained and controlled manner, resulting in accelerated healing (Hamdan et al., 2017). Furthermore, they enhance therapeutic efficacy, prolong release, improve patient compliance by reducing the dose frequency, increase bioavailability, increase site selection, and reduce other unwanted biopharmaceutical attributes (Sharma et al., 2016).

Hence, the commercialization and patenting of nanopolyphenols has increased significantly. Over the last 3–4 years, hundreds of patents related to nanopolyphenols have been published (Rambaran, 2022). A steady increase in the number of patents filed with herbal nanoformulations has been recorded in recent decades because of their advantages in overcoming the drawbacks faced by conventional delivery systems (Rambaran, 2022). Curcumin, quercetin, carotenoids, paclitaxel, and silymarin nanoparticles are the most filed herbal-based nanoformulation patents. Along with nanoemulsion, nanodispersion, and nanoencapsulation of *Withania somnifera* and emulsified nanoparticles of Arbutin (Jadhav et al., 2014; Jadhav et al., 2017).

Here, we highlight the most recent nanotherapeutic approach in which Wound Healing therapeutics are endowed via phytoconstituents-based advances.

### 5.1 Metallic and metallic oxide nanomaterials for diabetic wound healing

#### 5.1.1 Silver nanoparticles (Ag NPs)

Silver is a well-known antibacterial agent generally used to treat burns and wound infections. Changes in bacterial cell walls, genetic material, and disruption of respiratory enzyme pathways are the causes of wound infections. Reactive oxygen species and inflammatory cytokines are released when bacteria form biofilms in chronic wounds, which are encased in a protective extracellular polymeric matrix. This results in persistent inflammation, prevents re-epithelialization, and triggers apoptosis.

Although silver has well-known antibacterial and anti-inflammatory properties, using too much of the metal might be harmful (Singla et al., 2017a). Hence, for efficient healing, it is necessary to determine the ideal and safe concentration of silver to employ in dressings.

With silver, Wound Healing is faster and more effective owing to its superior effectiveness toward multidrug-resistant and biofilm-forming bacteria. Generally, its salts are extensively used to treat chronic wounds and burns infected with microorganisms living within biofilms (Costerton et al., 1999; Hamdan et al., 2017). Despite its proven antimicrobial properties, the application of silver alone can sometimes cause cytotoxicity and oxidative burst induction, and cannot maintain a stable concentration, easily causing local aggregation and adverse reactions (Prasath and Palaniappan, 2019). This problem was counteracted by synthesizing AgNPs with high surface-to-volume ratios at low concentrations. A variety of silver nanostructure shapes and sizes have been investigated, and their antibacterial properties have been demonstrated in different ways (Adhya et al., 2014). Owing to their nanosized and increased surface area and AgNPs exhibit antibacterial properties. These physicochemical properties allow them to penetrate bacterial cells, disrupt membranes, and cause intracellular damage, therefore inhibiting bacterial growth (Kumar et al., 2018). Besides, AgNPs accelerate Wound Healing through the proliferation and migration of keratinocytes, which makes them a viable option for treating diabetic ulcers. Tian et al. explored the wound-healing activity of AgNPs in a rat model and reported rapid healing. Their studies have provided a positive direction for this novel approach (Tian et al., 2007). Additionally, plant-based synthesized AgNPs were also found to be potent wound healers (Table 2). In this context, Manikandan et al. biosynthesized AgNPs from *Caulerpa Scalpelliformis extract* and reported their biomedical application in diabetic cutaneous wounds (Manikandan et al., 2019).

**TABLE 2:** List of the most recently developed metallic and non-metallic nanoparticles for diabetic Wound Healing.

S. No.	Phyto nano formulation	Mechanism of action	Experimental model	Result
1	Biosynthesized AgNPs from *Parrotiopsis jacquemontiana* (Ali et al., 2022)	Protect tissue against oxidative damage, enhance epithelialization rate, and increase collagen deposition	Excisional rat wound model	Showed antifungal, antibacterial, antioxidant, anticancer, and Wound Healing effects at minute concentrations
2	Biosynthesized AgNPs using aqueous extract of *Hypericum perforatum* (Alahmad et al., 2022)	Antibacterial activity against both Gram (+) and Gram (−) bacteria	Wound contraction method using fibroblast line (ATCC^®^ PCS-201–012)	Efficient antibacterial agent, Wound Healing activity yet to be confirmed
3	Hydrogel film of AgNPs and methanolic extract of *Mentha piperita* (Mojally et al., 2022)	Antioxidant and anti-inflammatory activity, enhanced fibroblast activity during the angiogenesis process, and reduced fasting blood glucose level	An acute diabetic wound-healing model measures the mRNA expression of genes involved in the wound-healing process	Potent Wound Healing activity in diabetic rats
4	Green synthesis AgNPs from Curcuma longa loaded cotton fabric (Maghimaa and Alharbi, 2020)	Active proliferation and fibroblast growth	*In vitro* model using fibroblast lines (L929)	Potent Wound Healing activity, antibacterial
5	Biosynthesis of AgNP using Persicaria odorata leaf extract (Lubis et al., 2022)	Bactericidal specifically against MRSA.	*In vitro* Wound Healing study using HSF 1184, normal human fibroblasts	Biocompatible, possesses antibacterial and Wound Healing aptitude
6	Green Synthesized AgNPs from *Thespesia populnea* leaf Ethanolic Extract (Abdel Halim et al., 2024)	Free radical scavenger	--	A potent antimicrobial and antioxidant agent
7	Green Synthesized AgNPs from aqueous extract of Cuphea carthagenensis (Rather et al., 2022)	Disrupt the cell membrane of both Gram (+) and Gram (−) bacteria, and free radical scavenging	--	A potent Wound Healing agent, Nontoxic against RBCs, bactericidal, and antioxidant properties
8	Biosynthesized Pomegranate extract and Chemically synthesis AgNPs(Scappaticci et al., 2021)	Inducing fibroplasia, increased collagen deposition, and improved anti-inflammatory effect	Rat excisional wound model	GS AgNPs improved wound closure and Wound Healing, reduced cytotoxicity
9	Phyto fabricated *Punica granatum* AgNPs-Chitosan Hydrogel (Ragab et al., 2019)	Enhanced re-epithelialization, collagen deposition, and improved anti-inflammatory activity	Excisional wound model, human cell line (HFB4)	Significant chronic diabetic Wound Healing property, acceptable cytotoxicity
10	*Syzygium cumini* Cellulose- AgNPs(Singla et al., 2017b)	reduced inflammation, promoted angiogenesis, rate of collagen deposition, and neo-epithelialization	Wounded diabetic mice model	Antimicrobial, cytocompatible, and effective Wound Healing agent
11	Green synthesized AgNPs(Younis et al., 2022)	Enhanced angiogenesis, enzymatic antioxidant level, and reduced inflammatory mediators	STZ-induced diabetic rat models	Quicker wound closure
12	Chitosan-AgNPs(Shehabeldine et al., 2022)	Antibacterial against Gram (+) and Gram (−), Antifungal, Antibiofilm, Antioxidant	Static biofilm model, Human skin cell line	Rapid Wound Healing at nontoxic concentration
13	CMC-loaded green synthesized AgNPs hydrogel (Ruffo et al., 2022)	Inhibits collagenase and Myeloperoxidase activity	Human cell line Activation Test (h-CLAT)	Positively affected Wound Healing, antioxidant, anti-inflammatory, and antimicrobial activity
14	PVA-bacterial Cellulose-Ag Nano Hydrogel (Song et al., 2021)	By inducing Angiogenesis	Mice model	Enhanced Wound Healing, Antibacterial, and anti-inflammatory properties
15	Phyto-engineered AuNPs(Boomi et al., 2020)	Free radical scavenger	BALB/c mice model	Promising antibacterial, antioxidant activity, and enhanced Wound Healing in diabetic mice
16	Chitosan-based Ag–Cu hydrogel (Liu et al., 2022)	Biocompatible antimicrobial agent, anti-inflammatory, promoted angiogenesis	*S. Aureus* infected skin incision model in diabetic wounds and normal rats	Efficiently accelerated tissue repair
17	ZnO-NPs(Elsawy et al., 2022)	Showed bactericidal effect	Incision Wound model	Promoted Wound Healing
18	Pterocarpus marsupium extract/chitosan NPs loaded hydrogel (Manne et al., 2021)	Enhanced re-epithelization and granular tissue growth, improvement in collagen deposition	Incisional wound model	Effective in curing diabetic wounds, with antibacterial and anti-inflammatory activity
19	Astilbin liposomes and diclofenac sodium loaded pH sensitive Carboxymethyl chitosan and sodium alginate oxide hydrogel (Wu et al., 2025)	Biocompatible, anti-inflammatory, antibacterial, and homeostatic activity	Incision wound in diabetic mice	Promotes Wound Healing by increasing angiogenesis and anti-inflammatory mechanisms

Younis and colleagues examined the ability of cyanobacterial species to produce AgNPs and the wound-healing characteristics of the developed nanoparticles in diabetic animals (Younis et al., 2022). The cyanobacterium biosynthesized AgNPs were found spherical with a diameter range of 10–35 nm. The formed AgNPs displayed a decrease in epithelialization period, augmented collagen and the wound closure percentage, hydroxyproline, and hexosamine contents, which enhanced angiogenesis factors (HIF-1α, TGF-β1, and VEGF) in excision wound models. Furthermore, AgNPs exaggerated superoxide dismutase (SOD), catalase (CAT), and glutathione peroxidase (GPx) activities, and glutathione (GSH) and nitric oxide content and reduced malondialdehyde (MDA) levels. Topical applications of AgNPs reduced the inflammatory mediators, including IL-6, IL-1β, TNF-α, and NF-κB.

Furthermore, an injectable thiolated chitosan hydrogel loaded with NO donors and silver nanoparticles (AgNPs) was developed for successful diabetic wound treatment (Huang Z. J. et al., 2025). From which NO was released stably and sustainably responsive to reactive oxygen species (ROS) at the wound site. In the end, this combined strategy facilitates the reconstruction of epithelial structures at the wound site, thereby offering a promising solution for diabetic chronic wound healing. It accomplished effective antibacterial action, biofilm prevention, inflammation suppression, vascular repair, and better local blood circulation.

Shelar et al., green-synthesized AgNPs utilising aqueous extracts of *Tagetes erecta* (Marigold) and *Portulaca oleracea* (Purslane) (Shelar et al., 2025). To enhance their antibacterial and wound-healing properties, they were further combined with a polyherbal gel formulation. In comparison to 60% (untreated) and 95% (povidone iodine), the polyherbal gel, which contained extracts of *Ficus racemosa, Emblica officinalis, Curcuma longa, Carica papaya, Terminalia bellerica, Acacia catechu, and Aloe vera*, achieved 85% wound healing in diabetic rats by day 16. Combinations of AgNP and antibiotics achieved 90% healing. There was no change in HbA1c levels, suggesting glucose-independent repair. These results suggest that AgNP-polyherbal formulations, which increase antibacterial activity and assure tissue regeneration, may be a viable and successful treatment for DFU.

#### 5.1.2 Gold nanoparticles (AuNPs)

The metal precursor used to make gold nanoparticles (AuNPs) is thermostable, making it extremely stable and non-biodegradable. As bulk gold has been proven to be non-toxic and bio-inert, it is utilized in medicine.

A second widely used nanomaterial is gold nanoparticles, which are employed for tissue regeneration, angiogenesis, Wound Healing, gene transfer, cancer cell imaging, biosensors, and targeted drug delivery (Elahi et al., 2018; Bai et al., 2020). Due to their biocompatible nature, surface reactivity, antioxidant, and surface plasmon resonance, AuNPs are significant components for their use in therapeutic and diagnostic applications (Sibuyi et al., 2021). AuNPs have been shown to have diverse therapeutic effects against several infectious, metabolic, and chronic diseases (Sibuyi et al., 2021). To aid in Wound Healing, AuNPs prevent bacteria from ROS formation and act as antioxidants. Thus, AuNPs promote Wound Healing and collagen regeneration through their antimicrobial and antioxidative properties. Similarly, the anti-inflammatory and antiangiogenic properties of AuNPs encourage the release of proteins that are vital for wound healing (Lau et al., 2017; Vijayakumar et al., 2019). Wound Healing (Lau et al., 2017; Vijayakumar et al., 2019). Ponnanikajamideen et al. synthesized AuNPs with the leaf extract of *Chamaecostus cuspidatus*, also known as the insulin plant (Ponnanikajamideen et al., 2019). They investigated and reported that AuNPs act as dose-dependent free radical scavengers. Additionally, they observed that blood glucose and insulin levels were restored in the Wound Healing activity mice (Ponnanikajamideen et al., 2019) model. However, to achieve better activity of AuNPs, they must be incorporated with other biomolecules, as compared to silver, gold nanomaterials alone do not possess much more antimicrobial activity. Also, gelatin, collagen, and chitosan can easily be crosslinked for better activity (Akturk et al., 2016). However, AuNPs can suppress the growth of multidrug-resistant pathogens by binding to the DNA of bacteria, blocking their double helix from unwinding during replication, and inhibiting ATP synthase enzyme activity, thereby killing bacteria (Mihai et al., 2019). Likewise, Boomi et al. prepared AuNPs using an aqueous extract of *Acalypha indica,* and their studies observed that AuNPs are efficient antibacterial, antioxidant, and promising Wound Healing agents (Boomi et al., 2020). The antibacterial potential of AuNPs could be due to as they enter into bacterial cells and alter their membrane potential, and the energy metabolism of bacteria is inhibited as a result (Wang et al., 2018; Wang et al., 2018). Besides, AuNPs act as sensitizers and inhibit lipid peroxidation to prevent ROS formation and reduce inflammation, thus promoting Wound Healing. Some studies have found that AuNPs can significantly accelerate Wound Healing and reduce scarring when combined with other antibacterial drugs, either synthetic or plant-based (Wang et al., 2022).

Recently, Ye and colleagues developed co-delivery of hemoglobin-resveratrol (Hb-RES) nanoparticles and morphologically switchable Au nanowires in microneedles for synergistic diabetic wound healing therapy (Ye et al., 2025). In a diabetic mouse model induced by streptozotocin (STZ), the microneedle progressively degraded, and the Hb-RES nanoparticles synergistically worked to reduce hypoxia, scavenge reactive oxygen species, and prevent macrophage differentiation into pro-inflammatory M1 phenotypes. Au nanowires constantly catalyze glucose during this procedure in the presence of inherent glucose oxidase activity. Consequently, this research offers new perceptions on the long-term control of blood glucose levels during synergistic diabetic wound healing.

Besides, Au-cluster-modified Prussian blue (PB) nanospheres (PB-Au) as antibacterial nanoplatforms for diabetic wound healing were developed (Ren et al., 2025). The prepared PB-Au showed tunable peroxidase (POD)-like activity and supported both photostability and catalytic stability, along with accelerating diabetic wound healing. The PB-Au enzyme exhibited the bacterial biofilm destruction of almost 86%, and the rate of bacterial eradication was more than 95%. According to Western blot (WB) results, PB-Au amplified the expression of platelet endothelial cell adhesion molecule-1 (CD3-1) and vascular endothelial growth factor (VEGF) by around 1.4 and 1.3 times, respectively.

#### 5.1.3 Copper nanoparticles (CuNPs)

Copper can cause apoptotic cell death and diminution in metalloproteinase gene activity, which reduces the inflammatory reactions and paces up wound healing (Zhou et al., 2015; Feng et al., 2019). Besides, when copper nanoparticles are utilized to treat wounds, in hypertrophic and keloid scars, high levels of TGF-B gene expression decline, while IFN-Y gene expression increases in response (Sandoval et al., 2022).

Furthermore*, E. coli, P. aeruginosa, S. aureus*, and other multidrug-resistant bacteria and fungi that are frequently detected in diabetic ulcer infections are significantly inhibited by CuNPs (Zheng et al., 2024). To kill bacteria, CuNPs release Cu^2+^, which solidifies the bacterial enzyme’s structure and function (Chatterjee et al., 2014). Kumar and colleagues biosynthesized *Cissus arnotiana* extract containing CuNPs *and reported* them as a potential antibacterial agent, primarily against Gram-negative bacteria (Rajeshkumar et al., 2019). Also, copper can stimulate angiogenesis in wounds by promoting VEGF production and help to boost immunity by producing interleukin-2, which increases immunity (Bauer et al., 2005; Bauer et al., 2005). Although copper is effective against harmful microbes, its toxicity to cells is dose-dependent, which should be considered when designing formulations (Tiwari et al., 2014). Hence, precise dose optimization is crucial for therapeutic safety. Moreover, oxidation and cluster formation are also potential issues; therefore, stabilizers (e.g., chitosan) should be used to improve the stability of CuNPs (Vijayakumar et al., 2019). Zangeneh et al., synthesized CuNPs with the leaf extract of *Fulcaria vulgaris* and performed various *in vitro* and *in vivo* studies, including Wound Healing studies in rats via an excision wound model; their results confirmed that green-synthesized CuNPs were found efficient as antifungal, antibacterial, antioxidant and cutaneous Wound Healing agents (Zangeneh et al., 2019).

Besides, a dual drug-delivery micro/nanofibrous core-shell system of polycaprolactone/sodium sulfated alginate-polyvinyl alcohol (PCL/SSA-PVA), engineered by the emulsion electrospinning method, was used to enhance the sustained delivery of copper oxide nanoparticles (CuO NP) (Alizadeh et al., 2024). The CuO NP (0.8%w/w) scaffold discloses the maximum tube formation in HUVEC cells and upregulates the pro-angiogenesis genes (VEGFA and bFGF) expression with no cytotoxicity effects. This study strongly recommends the 0.8%w/w CuO NP-loaded PCL/SSA-PVA as an outstanding diabetic wound dressing with significantly enhanced angiogenesis and wound healing.

Also, copper carbonate nanoparticles (NPs) of an average size of 55 ± 16 nm showed a crystalline structure, and antibacterial tests confirmed enhanced inhibition zones against *Pseudomonas* spp.*, S. aureus,* and other bacterial strains (Aslam et al., 2025). The largest zone of inhibition (18.5 ± 1.05 mm) was observed at 12 mg/mL for *Pseudomonas spp*. In wound healing activity in diabetic mice, remarks revealed a complete wound closure in NPs treated mice by day 14 as compared to the control group (96.10% wound closure). Hence, these results advocate their potential in biomedical applications, particularly for treating diabetes and bacterial infections.

#### 5.1.4 Metal oxide nanoparticles

Zinc oxide, titanium oxide, cerium oxide, yttrium oxide, and other metal oxide nanoparticles have gained interest in medical applications because they contain important minerals for humans, and even minimum amounts of these elements often exhibit strong therapeutic activity. Owing to their biocompatibility and several therapeutic applications, including antimelanoma, antidiabetic, antibacterial, and anti-inflammatory properties, which have potential for Wound Healing, zinc oxide nanoparticles (ZnO NPs) are promising drug delivery carriers (Ezhilarasu et al., 2020). Furthermore, ZnO NPs are effective Wound Healing agents because they are highly resistant to bacteria and adhere to the wound site for longer periods, thereby stimulating healing. Since ZnO NPs possess antibacterial properties, they can be used in nanocomposites for Wound Healing and skin infection treatment by either promoting the migration of keratinocytes or disrupting the bacterial cell membrane. There are still several drawbacks to the use of ZnO NPs in Wound Healing, including their intrinsic toxicity, which requires further investigation. However, when ZnO NPs are combined with other polymers to form hydrogels, which are further infused with adipose stem cells, they exhibit optimal antimicrobial activity with minimal toxicity, also enhancing wound healing potential, and making them ideal for use in wound dressings or other formulations (Ramzan et al., 2023).

Bai and Jarubula proposed a green and eco-friendly method for the preparation of ZnO NPs using the leaf extract of *Nigella sativa* plant (Bai and Jarubula, 2023). The synthesized ZnO NPs were characterized with DLS and FESEM and displayed polydisperse types of ZnO NPs with an average particle size of 45 nm. Additionally, antidiabetic studies exhibited the recovery of insulin, glycogen, and blood glucose levels in diabetic mice treated with ZnO NPs. Further, they suggested that this work unlocked the opportunities for future studies in the advancement of new drugs for use in diabetic wound care during sports training. Furthermore, a novel silver-zinc oxide-eugenol (Ag + ZnO + EU) nanocomposite was synthesized to improve antimicrobial activity and promote wound healing (Nagaiah et al., 2025). Nanocomposite confirmed effective antimicrobial efficacy against wound-associated pathogens, comprising standard and clinical isolates of *Pseudomonas aeruginosa*, *Staphylococcus aureus*, and *Candida* albicans. *In vitro* scratch assays utilizing human keratinocyte cells established that the nanocomposite significantly augmented wound closure (with near-complete healing observed within 24 h), presented enhanced cell migration, and tissue regeneration. Moreover, in an *in vitro* assay, the nanocomposite exhibited potential antidiabetic properties by enhancing glucose uptake (up to 97.21%). Subsequently, the potential of *Gliricidia sepium* (Jacq.) Kunth. ex. Walp. Leaves zinc oxide nanoparticles hydrogel (GSL ZnONPs HG) for diabetic wound healing was studied (Wafaey et al., 2025). GSL ZnONPs HG reduced apoptosis, improved tissue regeneration, and controlled inflammation in diabetic wounds as confirmed by wound closure and morphology analysis. A substantial decrease in vascular cell adhesion molecule-1 (VCAM-1) and advanced glycation end products levels (AGEs), and a noteworthy rise in interleukin-10 (IL-10) and platelet-derived growth factor concentrations (PDGF) were observed. Hence, this study advocated the potential of GSL ZnONPs HG as a hopeful approach to augment diabetic wound healing.

Similarly, titanium oxide nanoparticles (TiO_2_ NPs), can also be employed *in vivo* and *in vitro* to accelerate the healing of wounds (Nosrati and Heydari, 2025). TiO_2_ NPs show promising biological functionality, encompassing anti-inflammatory, antioxidant, and antimicrobial properties, making them desirable for wound healing (Ziental et al., 2020; Ikram et al., 2021). Besides, TiO_2_ NPs can be altered through procedures, for instance, coating, doping, or surface functionalization, which can improve their biological properties (Diana and Mathew, 2022). Moreover, dressings and scaffolds integrating TiO_2_ NPs have been revealed to have a significant improvement in the wound healing process (Ghosal et al., 2019; Ismail et al., 2019; Elekhtiar et al., 2025). Also, Altememy and colleagues studied the healing impact of calcium alginate scaffold-loaded TiO_2_ NPs using *origanum vulgare* L., carvacrol, *hypericum perforatum* L., and hypericin on *staphylococcus* aureus-infected ulcers in diabetic rats. They concluded that because of their antibacterial and anti-inflammatory properties, the medicinal plants *Origanum vulgare* and *Hypericum perforatum* - particularly their active components hypericin and carvacrol - reduce inflammation and the microbial load on wounds in diabetic rats, ultimately leading to wound regeneration (Altememy et al., 2022).

In addition, cerium oxide nanoparticles (CeO_2_) NPs are known for their activity as free radical scavengers. Furthermore, due to the thermal stability, favorable mechanical properties, exceptional oxygen storage capacity, and high retention rate of conjugated enzymes, the utilization of CeO_2_ NPs exhibits incredible potential in wound healing (Chen et al., 2024). Ahmad et al. produced CeO_2_ nanoparticles using *Abelmoschus esculentus* extract, which showed effective antioxidant, antibacterial, and Wound Healing effects (Ahmed et al., 2021). A novel nano-based wound dressing containing chitosan nanoparticles encapsulated with green synthesized cerium oxide nanoparticles using *Thymus vulgaris* extract (CeO_2_-CSNPs) (Kamalipooya et al., 2024). The electrospun PCL/cellulose acetate-based nanofiber was prepared, and CeO2-CSNPs were integrated on the PCL/CA membrane by electrospraying. The *in vivo* diabetic wound healing experiment revealed that PCL/CA/CeO2-CSNPs nanofibers can significantly increase the repair rate of diabetic wounds by up to 95.47% after 15 days.

The next metal oxide is yttrium oxide nanoparticles (Y_2_O_3_ NPs), which are nontoxic to neutrophils and macrophages. In addition, these NPs are mostly considered for the treatment of diabetic ulcers since they require the highest free energy compared with other metal oxides. CeO_2_ NPs and Y_2_O_3_ NPs act by protecting against oxidative stress damage because of their antioxidant properties. These nanoparticles reduce the production of ROS and prevent apoptosis (Vijayakumar et al., 2019).

### 5.2 Nonmetallic nanomaterials for diabetic wound healing

Among all the nonmetallic nanomaterials, carbon-based nanomaterials have been demonstrated tremendously for their potential application in diabetic wounds. They are proposed as curing agents and have several uses in nanomedicine, such as for tissue regeneration, bioimaging, and controlled drug delivery (Naskar and Kim, 2020). Recent studies reported the antibacterial and antifungal properties of graphene oxide nanosheets, making them effective against wound infections (Hamdan et al., 2017). Black phosphorous (BP) is also an emerging nanomaterial used in diabetic Wound Healing. Ouyang et al. demonstrated a study on BP-based *in situ* sprayed pain relief gel for treating diabetes wounds and provided an experimental-based application of BP in diabetic ulcer treatment. Xu et al. proposed the use of epigallocatechin gallate-modified black phosphorus quantum dots (EGCG-BPQDs@H) and demonstrated that they are promising multifunctional nanoplatforms for healing MRSA (methicillin-resistant *Staphylococcus aureus*)-infected burn wounds in diabetic patients (Xu et al., 2021). Naturally occurring polymers such as chitosan, alginate, and hyaluronic acid are also known for their antibacterial and rapid Wound Healing properties. Ribeiro et al. prepared insulin-containing chitosan NPs, and their report revealed that both blank and insulin-containing chitosan NPs were responsible for wound maturation (Ribeiro et al., 2020).

PLE-AgNPs can be synthesized efficiently by eliminating the need for hazardous reducing and capping agents. Additionally, PLE-AgNPs exhibit significant antioxidant and cell migration potential without cell cytotoxicity, indicating potential wound-healing properties (Sharma et al., 2024).

Compared with untreated cells, glucose uptake in 3T3-L1 cells was increased, glucose spikes were reduced, and Wound Healing in treated cells was significantly promoted. In 3T3/L cells, the AgNPs also demonstrated remarkable potential in accelerating Wound Healing, achieving 92% closure of wounds after 48 h of incubation (Majeed et al., 2024).

The synthesis of drugs based on medicinal or combined Co-ZnO NPs with greater targeted activity, synthesized from *C. officinalis* flowers, may lead to opportunities for the discovery of a less expensive and more beneficial therapy for Wound Healing (Aydin Acar et al., 2024).

Silver nanoparticles (AgNPs) were synthesized via a green method involving cucumber pulp extract. Ointment prepared with green synthesized AgNPs effectively healed wounds within 15 days while also exhibiting antibacterial and antioxidant properties (Iqbal et al., 2024).

Compared with the control group, the A. O-ZnO-NP group presented reduced downregulation of IL-6, IL-1β, and TNF-α and increased IL-10 levels, confirming the improved anti-inflammatory effect of the self-assembly method. *An in vivo study* and histopathological analysis revealed the superiority of the nanoparticles in reducing signs of inflammation and wound incisions in a rat model (Elhabal et al., 2024). Rutin-NPs have the potential to enhance the wound-healing process by attenuating oxidative stress, as evidenced by the restoration of GSH, CAT, and SOD antioxidants and decreased MDA production mediated by Nrf2 activation (Naseeb et al., 2024). The RW-AuNPs were found stable in the test solutions and showed no cytotoxicity to the KMST-6 cells for up to 72 h. AuNPs synthesized from Pinotage and Cabernet Sauvignon enhanced the proliferation of KMST-6 cells and showed potential as Wound Healing agents (Mgijima et al., 2024). Folic acid-decorated nanoparticle loaded with chitosan-gelatin hydrogel was prepared by researchers to overcome issues like poor bioavailability for topical applications. The structural characterization of the nanoparticles and the final gel was performed using TEM and SEM studies, respectively. These studies revealed that the nanoparticles enhanced Wound Healing, as demonstrated by cell migration assays, indicating that they could facilitate Wound Healing by promoting epithelialization (Bhardwaj and Jangde, 2024).

### 5.3 Nanomaterials used as carriers of therapeutic agents in wound healing

Nowadays, therapeutic drugs are delivered to specific locations and aid in Wound Healing processes using nanocarriers. Most chronic diabetic wounds that do not heal are infected with biofilm-forming microorganisms. Because bacteria produce a complex extracellular matrix that decreases the therapeutic response of conventional drug formulations, a conventional antibiotic therapy may not be enough to deliver the drug to the infected site (Naskar and Kim, 2020). Consequently, a method to functionalize drug-loaded nanocarriers to make them more effective in penetrating the biofilm matrix and target bacteria within it has garnered a lot of interest.

The potential advantages of nanocarriers include improved drug therapy and the capacity to modify a drug’s pharmacodynamic and pharmacokinetic characteristics holistically without altering its molecular structure. In addition to targeted delivery, they also release drugs at an optimal concentration in a controlled manner and protect against enzymatic degradation (Sivadasan et al., 2021). Among the most extensively employed nanocarrier systems for drug delivery are polymeric lipid hybrid nanoparticles, liposomes, and peptide nanostructures (Table 3). These have surfaced as a cutting-edge method for delivering drugs that exhibit antibiofilm properties. Their capacity to encapsulate and deliver both hydrophilic and lipophilic drugs concurrently, along with their simple design and preparation, biocompatibility, and biodegradability, has facilitated their use as drug delivery carriers in a range of therapies, including Wound Healing, to accurately deliver at the target site (Wilhelm Romero et al., 2021).

**TABLE 3:** Various nanoformulations for Wound Healing.

S. No.	Phyto-nano formulations	Polymer/s used	Experimental model	Results
1	NPs loaded with Quercetin in hydrogel matrices (Badhwar et al., 2021)	Carbopol-934	Excisional diabetic wound model	Superior activity against *S. aureus* and *E. coli*. Reduced wound gap and increased re-epithelization
2	*ZnO-NPs by Althaea officinalis extract* (Elhabal et al., 2024)	Chitosan hydrogel	*In vivo* study using male Albino wistar rats	Improved anti-inflammatory activity and wound healing potential
3	Chitosan and gelatin-based biocomposite films of green-synthesized AgNPs(Gupta et al., 2022)	Chitosan and gelatin	*In vitro* study using mouse fibroblast line (L929)	Antibacterial, promoted fibroblast migration, hence facilitated Wound Healing
4	PVA/chitosan/oxalic acid nanocomposite hydrogels loaded with AgNPs(Popescu et al., 2022)	Chitosan and Polyvinyl alcohol	Human dermal fibroblast	Antimicrobial, acceptable cytotoxicity, and efficient wound healer
5	*Aloe vera*/*Gum Arabic*/Ag nanocomposites (Naveen et al., 2022)	Gum Arabic	*In vitro* scratch Wound Healing activity using NIH3T3 cells	Biocompatible, excellent antibacterial, and promising Wound Healing agent
6	*Centella aisatica* extract mediated ZnO nanoparticles (Wang et al., 2024a)	Poloxamer	*In vitro* evaluation in Mice fibroblasts, Skin fibroblast and HUVEC cell lines	Good biocompatibility, cell proliferation, and tube formation potential
7	*Piper cubeba* oil nano emulsion (self-emulsifying) (Shakeel et al., 2019)	*--*	Excisional wound model in rats	Nontoxic, increased collagen deposition, enhanced Wound Healing
8	*Propolis* containing microemulsion (self-emulsifying) (Marquele-Oliveira et al., 2019)	Bacterial cellulose membrane	*In vitro and* *In vivo* Wound Healing model	Antibacterial, anti-inflammatory, and promote Wound Healing
9	*Moringa oleifera* extract loaded Phytosome (Lim et al., 2019)	-	Human dermal fibroblast	Nontoxic, potent Wound Healing agent
10	*Malva sylvestris extract containing nanofiber* (Almasian et al., 2020)	Carboxymethyl cellulose and Polyurethane	Wound Healing model using male Wistar rats	Antibacterial effect, promoted collagen deposition and neovascularization, and efficient diabetic Wound Healing activity
11	*Lithospermi radix* Extract/gelatin/fish collage loaded dual layer Nanofiber Scaffold (Yao et al., 2019)	Gelatin & Chitosan	*In vitro* study using mouse fibroblasts (L929) and *In vivo* study by excisional wound model in rats	Nontoxic, potent Wound Healing agent
12	*Polyherbal extract incorporated-Guar gum/PVA-based scaffold* (Kalachaveedu et al., 2020)	Guar gum and polyvinyl alcohol	*In vivo,* a Wound Healing model using male Wistar rats	Cytocompatibility, increased expression of stem cells, and the gene responsible for Wound Healing reduce scarring
13	Multi-functional iron modified ceria nanoparticles-integrated *astragalus* polysaccharide hydrogel (Zhang et al., 2025)	*Astragalus* polysaccharide (APS), polyvinyl alcohol (PVA)	*In vitro* study in macrophages and HUVEC cells *In vivo studies* in the STZ-induced mice model	Enhanced tissue regeneration, reduced apoptosis, and modulated inflammation

### 5.4 Nanomaterial-based scaffolds for diabetic wounds

Designing and developing scaffolds loaded with various therapeutic agents is a significant approach for diabetic wound therapy and is commonly employed in wound care. It involves the use of either natural or synthetic biomaterials, for instance, composites and hybrids. They not only allow adequate air and moisture permeability but also provide proper cell migration and proliferation, along with protection from external contamination and microbial invasion. Polymeric biomaterials with integral properties of Wound Healing have been reported and are the most promising choice for wound care management (Ijaola et al., 2022). Many microparticulate and nanoparticle-based systems, hydrogels, and fibrous scaffolds derived from nanotechnology have demonstrated great potential as wound-healing materials (Negut et al., 2020).

A scaffold is comprised of various polymers and is typically a short-lived framework. Numerous studies have demonstrated the use of biodegradable polymers in the making of 3D-bioprinted scaffolds for tissue engineering, including both natural (for example, collagen, chitosan, and gelatin) and synthetic (for instance, polycaprolactone, poly (lactic-co-glycolic acid), and polyethylene glycol) compounds. Synthetic polymers often exhibit more robust mechanical properties than natural polymers, even though natural polymers are usually extremely biocompatible. The most suitable scaffold for tissue regeneration is the original matrix of the target tissue in the original tissues, which performs numerous tasks, has a complex composition, and plays a vital role in determining the physiological identity of the tissue. Therefore, scaffolding is currently attempting to mimic the functions of the original extracellular matrix to the greatest extent possible (George and Pragna, 2022).

Wang et al. developed hyaluronic acid and natural silk fibroin-based scaffolds, and their results indicated better cytocompatibility, proliferation, and differentiation as well as avert scar formation (Wang Q. et al., 2021). Furthermore, Fu et al. prepared a composite scaffold of curcumin and poly (ε-caprolactone)-PEG-poly (ε-caprolactone), and the resulting scaffold enhanced Wound Healing (Fu et al., 2014). Natrajan et al. developed a biodurable porous scaffold of collagen using tannic acid as a cross-linking agent through a casting technique, and their results indicated increased wound closure and Wound Healing rates (Natarajan et al., 2013).

Besides, Metwally et al. developed a bioinspired 3D-printed scaffold embedding DDAB-nano ZnO/nanofibrous microspheres for regenerative diabetic Wound Healing (Metwally et al., 2023). Multiple assessments observed that the treatment of *Staphylococcus aureus*-infected full-thickness diabetic wounds in rats showed the superiority of DZ-MS@scaffold. The scaffold showed 95% wound-closure, effective regulation of healing-associated biomarkers, infection suppression, together with regeneration of skin structure in 14 days.

Additionally, Ma and colleagues produced a novel multifunctional self-assembled nanocellulose-based scaffold for the healing of diabetic wounds (Ma et al., 2025). In this, a nanocellulose-based smart scaffold with silk fibroin-loaded cerium oxide was developed for the treatment of diabetic wounds. Smart scaffold dressing displays excellent porosity, water retention, water absorption, controlled degradability, air permeability, and antioxidant properties. *In vitro* experiments demonstrated antibacterial activity against both Gram-positive (*S. aureus*) and Gram-negative (*E. coli*) bacteria. The *in vivo* results displayed that smart scaffold dressing can reduce inflammation at the wound site of diabetic mice and promote collagen deposition, angiogenesis, and re-epithelialization during Wound Healing in diabetic mice, demonstrating promising biocompatibility and biodegradability.

### 5.5 Natural polymers based dressings for diabetic wound healing

The wound dressing market currently offers a variety of products, including antimicrobial ointments, creams, gels, *etc.*, combined with natural biodegradable proteins and polymers such as cellulose, chitosan, collagen, hyaluronic acid, silicon, and gelatin. These naturally occurring polysaccharides have been widely used in the production of different products for wound management, as they bio-mimic and recreate the native extracellular matrix to a great extent. In addition, most of these compounds possess intrinsic anti-inflammatory and antibacterial properties. A list of various natural polymers that can be employed in the treatment of diabetic Wound Healing is represented in Table 4.

**TABLE 4:** Application of natural polymers in enhancing diabetic Wound Healing.

Natural polymers	Wound healing property	Available products
Collagen (Alberts et al., 2025)	The principal structural component of the extracellular matrix, mediates platelet aggregation, cell adhesion	• PuraPly AM • Diacoll-S • Systagenix • Hydrofera Blue • Hollister • Aquacel Ag • Medline • Collatamp®E.G.,
Cellulose (Bukatuka et al., 2025)	Entrap wound discharge due to high water absorption potential, stimulates Platelet-derived growth factor, Fibroblast growth factor, and Epidermal growth factor which increases granulation tissue formation and vascularization	• Fibdex • Cutimed sorbion • Surgiclean • aquaRite • Suprasorb • Nexoseal
Chitin/Chitosan (Wang et al., 2021c)	Oxygen permeability, improves fibroblasts, macrophages, inflammatory cell functions, antimicrobial activity, Rapid bone regeneration at initial stages, and enhances wound granulation, its degradation products take part in the makeup of the ECM and cartilage	• *Axiostat* • *Maxiocel* • *Anscare* • *Dual stop V* • *Chitosan 100* • *Kytocel*
Gelatin (Irfan et al., 2022)	Partially hydrolyzed derivative of collagen which facilitates nutrient transport, maintains molecular signal, and improves cell growth and proliferation	• Hemosponge • Gelspon • Surgispon • Surgifoam • Abgel • Flogel
Alginate^a^	It has good gelling properties, the ability to absorb fluids, maintain wound moisture, promote granulation tissue formation, and stimulate monocytes to produce an increased level of cytokines	• Stayguard • Algisite-M • AlgiDerm • Sorbsan • Kaltostat • Omiderm
Hyaluronic acid (Antoszewska et al., 2024)	Forms a smaller part of the ECM, promotes the formation of a fibrin clot, stimulates fibroblast proliferation, releases chemokines, and differentiates fibroblasts into myofibroblasts	• Hyalosafe • Hyalomatrix • Hylase wound gel • Hylasponge • Hyalofill • Bionect • Laser skin

^a^ https://www.woundsource.com/product-category/dressings/alginates Accessed on 25–06-2025.

## 6 Translation of therapeutic approaches for diabetic wounds

### 6.1 Patents for diabetic wound healing

Several patent studies related to phytopharmaceutical agents have been reported, but to the best of our knowledge, no studies related to nanoherbal formulations are currently available. The Patent US10206886B2, Lipid nanoparticles for Wound Healing using epidermal growth factor, and EP2895209B1, improved Wound Healing compositions comprising microspheres of polystyrene, the only nanoformulations for Wound Healing that have been patented to date. However, the pipeline for phyto/nano therapeutics plays a vital role. Nanobased topical medicines are safe and easy to use in the clinic when they are derived from phytochemical nanoformulations for the treatment of diabetic wounds. Some of the recently patented herbal formulations for treating wounds are listed in Table 5.

**TABLE 5:** List of currently patented formulations or phytoconstituents effective in treating diabetic wounds.

S. No.	Title	Indication	Patent No.
1	Topical composition with active *abis sativa* and *Calendula officinalis* for reduction of skin lesions	Treat skin lesions	US11364273B2
2	Multifunctional formulation comprised of natural ingredients and method of preparation/manufacturing thereof	Treatment for the compromised tissue	US20210346456A1
3	Methods of accelerating Wound Healing using cannabinoid compositions	Epidermal Wound Healing	US20200376156A1
4	Herbal preparation for accelerating wounds and skin inflammation healing, especially for the treatment of herpes and acne, and its application	Reduced inflammation and promote Wound Healing	US20190183954A1
5	Botanical formulations (using extracts of *Arctium lappa *root*, Epilobium angustifolium*, and *Cystoseira amentacea*)	Dermal remodeling, reduces skin lesions and acne	US9561198B2
6	Phyto complexes exhibiting multiple synergistic antioxidant activities useful in food, dietary supplements, cosmetic preparations, and pharmaceutical preparations	Antioxidant, tissue modulator	KR20180021674A
7	Pharmaceutical compositions comprising *arrabidaea chica* extracts in controlled release systems, production process and use thereof	Wound Healing	WO2013091056A1
8	Herbal Combinations for Wound Healing in Fibroblasts (including *rheum tanguticum,* *rehmannia glutinosa,* and *lonicera japonica*)	Enhanced Wound Healing by fibroblast migration	US20180185428A1
9	Herbal preparation for accelerating wounds and skin inflammation healing and its application (using *Melittis melissophyllum extract*)	Reduced inflammation and promoted Wound Healing	US20180318375A1
10	A topical herbal healing formulation (comprising *Centella asiatica, Plantago major, Scrophularia nodosa, Achillea millefolium and Tabebuia impetiginosa*)	Anti-inflammatory, antibacterial, active in Wound Healing but not in eczema and rashes	US20200330543A1
11	Biomimetic pro-regenerative scaffolds and methods of use thereof	Polymeric fiber scaffold for Wound Healing	US20200376170A1
12	Lipid nanoparticles for Wound Healing	Promote Wound Healing	US10206886B2
13	Ointment for Wound Healing (using *Hypericum perforatum*, *Veronica officinalis*, leaves and stems of *Lilium candidum*, *etc.*)	Polyherbal formulation with high Wound Healing effect	RU2736214C1
14	Improved Wound Healing compositions comprising microspheres	Fasten Wound Healing and wound closure	EP2895209B1
15	Compositions comprising extracts of Boswellia, tea tree, aloe and lavender oil and methods of treating wounds, burns and skin injuries therewith	Heal tissue injury	US20150030708A1
16	Antibacterial composition based on natural plant raw material and application of antibacterial composition	Effective in wound infection	CN104688810A
17	Antimicrobial silver and acemannnan composition for the treatment of wounds or lesions or burns	Treat wound lesions	EP 2704729B1
18	Processed *hyoscyamus* seed agent for the treatment of tissue disruption	Wound Healing	WO2006116804A1
19	Research method for promoting healing of diabetic wound by ginger extract hydrogel	Wound healing	CN 202411901886 A
20	Nano poly (lactic-co-glycolic) acid encapsulated tinospora cordifolia and uses thereof	Wound healing	IB 2024057244 W

^a^ Accessed from https://www.freepatentsonline.com/on 17 June 2025.

### 6.2 Clinical trials related to diabetic wound healing

Although Wound Healing treatments have improved in recent years, no single treatment works for all types of wounds. Therefore, scientists are always searching for novel medications and dressings that can work on injured tissue to promote recovery. Many phytochemical-based therapies, for instance, nanoparticles loaded with curcumin, *Centella asiatica*, berberine, *Plectranthus amboinicus*, honey, *etc.*, have been investigated in multiple clinical trials, despite the difficulty of conducting clinical trials with nano-based phyto-formulations. The list of ongoing/completed clinical trials using nanoparticles or nano-herbal formulations to treat diabetic wound ulcers is presented in Table 6.

**TABLE 6:** Clinical trials for plant extract and/or nanoparticles for diabetic wound healing.

S. No.	Clinical study id	Title	Interventions	Study details
1	NCT05243810	EPC Silver Wound Gel (EPC-123) feasibility study in the management of mildly infected diabetic foot ulcers	Device: EPC Silver Wound Gel	To evaluate the clinical safety and impact of EPC Ag gel on wound infection, wound ecology, and immunological biomarkers, to clarify parameters for a potential future study
2	NCT04834245	Evaluation of Diabetic Foot Wound Healing Using Hydrogel/Nano Silver-based Dressing vs Traditional Dressing	Procedure: Hydrogel/nano silver-based dressing	Compare the effectiveness of using hydrogel/nano silver-based dressing vs traditional dressing on diabetic foot Wound Healing
3	NCT04962139	Evaluate the Safety and Efficacy of ON101 Cream for the Treatment of Chronic Diabetic Foot Ulcers	Drug: ON101 Cream Other: Vehicle Cream	ON101 Cream contains *Plectranthus amboinicus* and *Centella Asiatica* extracts
4	NCT05338463	Fespixon Cream for the Treatment of Chronic Diabetic Foot Ulcers (TEXAS 1A, 2A) in Dialysis Patients	Drug: Fespixon Cream	Active ingredients- *Plectranthus amboinicus* extract (1.25%) and *Centella asiatica* extract (0.25%)
5	NCT01070433	A Clinical Study of the Safety and Efficacy of MEBO Wound Ointment in Subjects with Diabetic Foot Ulcers	Drug: MEBO Wound Ointment (MEBO) Other: Standard of Care	Phase II, randomized, controlled, multicenter study designed to assess the safety and efficacy of MEBO in the treatment of subjects with DFUs
6	NCT01154374	A Clinical Study of the Safety and Efficacy of MEBO^®^ Wound Ointment in Subjects with Diabetic Foot Ulcers (Pilot)	Drug: MEBO Wound Ointment Procedure: Standard of Care (sterile saline moistened gauze)	Phase II, randomized, controlled, 2-center pilot study designed to assess the safety and efficacy of MEBO in the treatment of subjects with DFUs
7	NCT03934281	Study of the Value of Using a Honey Dressing Compared to the Use of a Standard Dressing on the Toe Amputation Wound in the Diabetic Patient (MELIDIAB)	Device: Honey dressing Melectis G Device: HAS recommendation dressing	To compare the rate of epidermisation at 6 months for these wounds, between honey dressing and other dressing devices used
8	NCT03649243	Propolis as Adjuvant in the Healing of Human Diabetic Foot Wounds	Drug: Propolis spray	Observational study
9	NCT04634838	Efficacy of Wound Dressings with Copper Oxide	Device: MedCu Antibacterial Wound Dressings with Copper Oxide	Comparison between the efficacy of antibacterial wound dressings containing CuO microparticles to improve the Wound Healing of pressure sores and postop wounds as compared to Ag wound dressings
10	NCT00971048	Evaluation of the Effects of HP828-101 Versus Standard of Care in the Management of Partial or Full Thickness Wounds	Device: HP828-101|Device: Hydrogel/Hydrocolloid	To compare HP828-101 to a standard of care for the management of partial or full thickness wounds, wound closure by day 22, comparison of pain assessed, and evaluation of moist wound environment
11	NCT04963998	Safety and Efficacy Study of Med Cu Wound Dressings	Device: Application of Wound Dressings with Copper Oxide	Application of Wound Dressings with Copper Oxide on chronic foot wounds in diabetic patients
12	NCT01427569	Efficacy Study of IZN-6D4 Gel for the Treatment of Diabetic Foot Ulcers	Drug: IZN-6D4 Gel Other: Placebo hydrogel	Standard wound therapy plus twice weekly topical application of hydrogel containing botanical extracts
13	IRCT20201213049703N1	Effect of herbal ointment in treatment of ulcers diabetes	herbal ointment of thirsty plant extract, honey, black seed oil, mazo	Once a day using sterile gauzes and adhesive until complete treatment

^a^ Accessed https://clinicaltrials.gov/on 16 May 2025.

## 7 Conclusion

Over the past few years, diabetic wound management research has grown exponentially, as evident by the experimental research available in databases, but its clinical translation remains very poor, as discussed earlier. A common consequence of diabetes is impaired Wound Healing, which can have devastating consequences for patients suffering from the disease. As the cases of chronic diabetic wounds have become a major burden in recent times. This has led the USFDA to launch special programs focusing on the accelerated development of therapies for Diabetic Wounds. Even after these efforts, the therapy for Diabetic wounds is a limited success. Various studies suggest that diabetes affects Wound Healing by different mechanisms. A variety of approaches have been evaluated but with limited success, including stem cells, growth factors, cytokine modulators, antimicrobial and anti-inflammatory drugs, matrix metalloproteinase regulators, angiogenic stimulators, extracellular matrix promoters, and several phytopharmaceuticals. The ability to extricate the extent of healing can be improved with better delivery systems and clinical methodologies. Hence, researchers must work on the latest developments in the design of novel carriers and gain a deeper understanding of the basic concepts involved in the successful translation of existing approaches in clinical settings. The management of compromised diabetic wounds can be treated better by combining approaches. Therefore, the application of nanotechnology and herbal therapeutic agents can overcome obstacles associated with conventional delivery systems and diabetic wound complications simultaneously.
